# Transport of lipophilic carboxylates is mediated by transmembrane helix 2 in multidrug transporter AcrB

**DOI:** 10.1038/ncomms13819

**Published:** 2016-12-16

**Authors:** Christine Oswald, Heng-Keat Tam, Klaas M. Pos

**Affiliations:** 1 Institute of Biochemistry, Goethe University Frankfurt, Max-von-Laue-Strasse 9, D-60438 Frankfurt am Main, Germany

## Abstract

The deployment of multidrug efflux pumps is a powerful defence mechanism for Gram-negative bacterial cells when exposed to antimicrobial agents. The major multidrug efflux transport system in *Escherichia coli*, AcrAB–TolC, is a tripartite system using the proton-motive force as an energy source. The polyspecific substrate-binding module AcrB uses various pathways to sequester drugs from the periplasm and outer leaflet of the inner membrane. Here we report the asymmetric AcrB structure in complex with fusidic acid at a resolution of 2.5 Å and mutational analysis of the putative fusidic acid binding site at the transmembrane domain. A groove shaped by the interface between transmembrane helix 1 (TM1) and TM2 specifically binds fusidic acid and other lipophilic carboxylated drugs. We propose that these bound drugs are actively displaced by an upward movement of TM2 towards the AcrB periplasmic porter domain in response to protonation events in the transmembrane domain.

## Abstract (web summary)

The AcrB module of the AcrAB-TolC multidrug efflux pump sequesters drugs from the periplasm and outer leaflet of the inner membrane. Here, Oswald *et al*. provide evidence that lipophilic carboxylated substrates bind to a groove between transmembrane helices TM1 and TM2, for further transport by an upward movement of TM2.

Bacteria deploy several mechanisms to combat invasion of antimicrobial compounds^1^. One of the first defence mechanisms is to avoid the accumulation of these toxic agents inside the cytoplasm or periplasm to concentrations lethal to the cell. In Gram-negative bacteria, downregulation of porin production is often used to slow down the antibiotic entry into the periplasm or cytoplasm^2^. In addition, upregulation of multidrug efflux pumps increases the effect of reducing the drug concentration in these compartments^3^. In Gram-negative bacteria, many of these transporters are part of so-called tripartite systems that allow the extrusion of substrates across the entire cell envelope, including the transport across the outer membrane^4^. The AcrAB–TolC complex from *Escherichia coli* is the model tripartite transporter system. The inner membrane protein AcrB is a member of the Resistance Nodulation cell Division (RND) transporter superfamily and uses the proton motive force to expel drugs in a drug/H^+^ antiport manner across the membrane. It forms a complex with the periplasmic membrane fusion protein AcrA and the outer membrane factor TolC. AcrB recognizes a wide variety of substrates covering various classes of hydrophobic antibiotics, dyes, detergents, organic solutes and even bile salts^5^,^6^, but also carboxylated drugs such as fusidic acid and β-lactams.

Within the homotrimeric setup, each AcrB protomer comprises a large periplasmic domain and a transmembrane (TM) domain with 12 TM-spanning helices (TM1–12) that form a 10-helix bundle around 2 core helices TM4 and TM10 (Fig. 1). The latter two helices contain three essential residues (D407, D408 and K940, another essential residue, R971, resides on TM11) important for H^+^ binding, H^+^ transport and energy transduction^7^,^8^,^9^. The TM domain consists of structural parallel repeats, R1 and R2, each comprising five helix bundles. The amino-terminal repeat, R1, includes TM1 and TM3 to TM6, while the carboxy-terminal repeat, R2, comprises TM7 and TM9 to TM12 (Fig. 1)^10^. Each repeat is flanked by a single TM helix that seems to function as a coupling element with the periplasmic porter domain (Fig. 1). The flanking helices are TM2 and TM8, respectively; TM2 is linked to the flexible PN2/PC1 repeat in the porter domain, while TM8 is connected to the more rigid PN1/PC2 unit (Fig. 1). The large periplasmic part, subdivided into a porter and a funnel domain, consists of two loops located between TM1 and TM2, and between TM7 and TM8 and comprises structural repeats of two similar α/β subdomains (PN1, PN2, PC1 and PC2) joined by a common β-strand (Fig. 1).

On basis of structural and functional studies, the drug specificity filter of the AcrAB–TolC efflux pump resides in the AcrB periplasmic porter domain (Fig. 1)^11^,^12^,^13^. Several drug substrates of AcrB have been shown to bind to two areas within the porter domain, known as the access pocket and deep binding pocket^14^,^15^. Structures of asymmetric AcrB^14^,^15^,^16^,^17^,^18^,^19^ revealed binding of minocycline (pdb entries: 2DRD^16^, 3AOD^15^, 4DX5^14^), doxorubicin (pdb entries: 2DR6 (ref. 16), 4DX7 (ref. 14)), erythromycin (pdb entry: 3AOC^15^), rifampicin (pdb entries: 3AOB, 3AOD^15^), rhodamine 6G (pdb entry: 5ENS^20^), or pyridopyrimidine/pyranopyridine inhibitors (pdb entries: 3W9H^19^, 5ENO, 5ENP, 5ENQ, 5ENR^20^) in either or both the access and deep binding pockets in the porter domain (Fig. 1b). Both periplasmic binding areas are situated along putative drug transport pathways comprised by the individual functional states L, T and O. Current hypothesis describes that these three states can be adopted by any of the protomers constituting the AcrB trimer. That is, when the trimer is actively pumping, the AcrB protomers cycle through the three states L, T and O^17^ (or access, binding, extrusion^16^) in a consecutive manner. It has been proposed that high molecular weight drugs (like rifampicin, erythromycin and doxorubicin dimers) are taken up from the periplasm via the access binding site in the L state from where they can reach the deep binding pocket upon transition to the T state towards the interior of the porter domain^14^,^15^. Low molecular weight drugs might enter the periplasmic domain via direct pathways towards the deep binding pocket^15^. Upon the energy-dependent T to O transition, the deep binding pocket collapses, squeezing drugs out, which are subsequently guided through an exit tunnel towards the TolC channel. While this mechanism only explains sequestering of drugs from the periplasmic space, it has been postulated for drugs located in the *E. coli* cytoplasm, that single component drug /H^+^ antiporters like EmrE^21^ and/or MdfA^22^ translocate the drugs to the periplasm from where these can be further transported by the tripartite RND system as described above^23^. For specific drugs like fusidic acid and β-lactams, which partition into the outer leaflet of the inner membrane^6^, however, no other *E. coli* transporter except RND-type transporters have been shown to be involved in the resistance phenotype^24^ and their transport route via AcrB is elusive.

Here, we present an asymmetric structure of AcrB in complex with fusidic acid. Substrate binding appears to be mediated by a groove constituted by TM1 and TM2. This groove is pseudosymmetric to the TM7/TM8 groove of the same protomer, the latter postulated to be a binding site for drugs from within the outer leaflet of the inner membrane^25^ and is located proximal to the predicted tunnel entrance leading to the deep binding pocket^5^,^18^.

In this report, substitution analysis by site-directed mutagenesis of those residues comprising the putative fusidic acid binding site in the TM1/TM2 groove render the cells harboring the variant AcrB pumps less effective against fusidic acid and other hydrophobic substrates containing carboxylic acid moieties. Moreover, fusidic acid and dicloxacillin could effectively protect the single cysteine AcrB variant C338 against cross-linking with sulforhodamine methanethiosulfonate (MTS-rhodamine) in a concentration-dependent manner, supporting the role of TM1/TM2 as binding site for these drugs. From the results presented here, we hypothesize that movement of transducing helix TM2 perpendicular to the membrane plane during functional protomer cycling, facilitates the displacement of fusidic acid towards the AcrB porter domain.

## Results

### Binding of fusidic acid to the TM1–TM2 region

Well-diffracting co-crystals of AcrB/DARPin in complex with fusidic acid were obtained under conditions with 100-fold molar excess of this compound over AcrB. The wild-type AcrB structure (pdb entry: 4DX5) was refined against the 2.5 Å native data set with resulting *R*/*R*_free_ of 22.5 and 26.2, respectively (Supplementary Table 1). The electron densities derived by simulated annealing composite omit procedure (to reduce model bias) displayed an additional unaccounted electron density (Fig. 2a) located next to TM1 (I27) and TM2 (K334, I337, H338 and V341) of the T conformer at a position ∼6 Å from the periplasmic side of the lipid bilayer. We assigned this electron density to a fusidic acid molecule (Fig. 2b).

Further inspection of the electron density maps in the same area of TM1 and TM2 in the L and O conformers led to identification of similar shaped, albeit less pronounced, electron densities. To obtain more distinct information of fusidic acid binding to the individual protomers, we co-crystallized the AcrB/DARPin complex with 24-bromofusidic acid (Supplementary Fig. 1a) and took advantage of the strong anomalous signal of the bromine atom at the K-edge (0.92 Å). We detected clear anomalous densities at the anticipated TM1/TM2-binding groove of the T and O protomers (Supplementary Fig. 1b,c) but no anomalous signal density at the L protomer. Overall, the *F*_o_−*F*_c_ densities of 24-bromofusidic acid were much weaker compared with that of the fusidic acid AcrB complex data set, indicating an overall less affine binding of 24-bromofusidic acid. However, the strong anomalous signals at the bromine edge clearly indicated the presence of the fusidic acid derivative at the T and O protomers located close to the C-24 of fusidic acid (Supplementary Fig. 1b,c), as expected. We also conducted a polder map calculation^27^ of the TM1/TM2 groove area. Here, clear densities are visible in the L and T protomer at the TM1/TM2 groove, whereas the density at the O protomer is not attributable to fusidic acid (Supplementary Fig. 2), in contrast to the clear binding observed for 24-bromofusidic acid (Supplementary Fig. 1c). As the density derived from the AcrB/fusidic acid complex and the 24-bromofusidic acid/AcrB complex data were complementary in the assignment of the bound molecules to the asymmetric AcrB trimer, we tentatively modelled one fusidic acid molecule per protomer within the asymmetric AcrB trimer structure (Supplementary Fig. 3). Based on the SA omit map and anomalous density analysis (Fig. 2 and Supplementary Figs 1 and 2), we consider the groove at the T protomer as the primary binding site, whereas the binding sites at the O and L protomers appear to have much less affinity for both fusidic acid and 24-bromofusidic acid.

**Figure 1:**
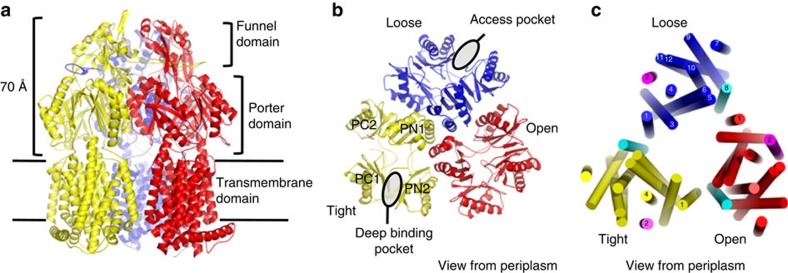
Overall structure of asymmetric AcrB trimer. (**a**) Side view of the overall structure of the asymmetric AcrB trimer depicts the loose (L), tight (T) and open (O) conformations in blue, yellow and red, respectively. The large periplasmic part protrudes ∼70 Å into the periplasm. It comprises the N-terminal PN1 and PN2, and the C-terminal PC1 and PC2 subdomains, and is divided in a porter domain and a funnel domain. (**b**) Top view from the periplasm on the porter domain. This domain consists of the PN1 and PN2 subdomains, originating from the N-terminal periplasmic loop between TM1 and TM2, as well as PC1 and PC2 subdomains, which originate from the C-terminal periplasmic loop between TM7 and TM8. The access pocket^14^,^15^ is situated between PC1 and PC2 subdomains in the L (blue) conformer. The deep binding pocket^16^,^17^,^18^ is located between PN2 and PC1 subdomains of the T conformer (yellow). (**c**) Top view representation from the periplasmic side of the asymmetric AcrB trimer transmembrane domain. TM1, 2–6 and TM7, 8–12 form a pseudosymmetric transmembrane core domain in each monomer (indicated in blue, yellow and red). TM2 and TM8, coloured in magenta and cyan, respectively, are considered energy-transducing helices from the transmembrane domain to the periplasmic porter domain and vice versa^10^.

**Figure 2:**
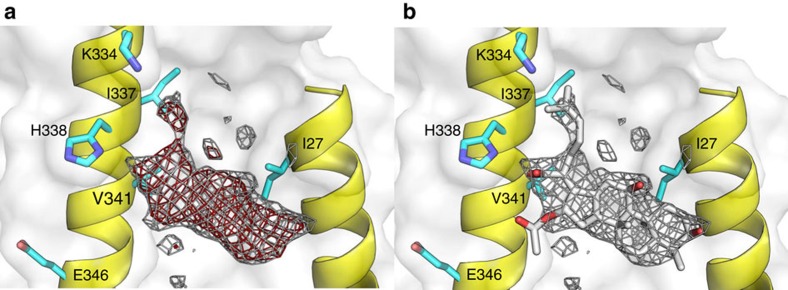
Observed *F*_o_−*F*_c_ and 2*F*_o_−*F*_c_ electron density assigned to fusidic acid located at the TM1-TM2 groove of the AcrB T protomer. (**a**) Simulated annealing (SA) composite omit 2mFo-DFc electron density (grey mesh, contoured at 1.2*σ*, 0.8167, e Å^3^) and SA composite omit mFo-DFc electron density (red mesh, contoured at 1.9*σ* (1.1086, e Å^3^) at the TM1/TM2 groove of the Tight protomer. (**b**) As in **a** with the assignment of a fusidic acid molecule (FUA, grey stick) in the SA composite omit 2mFo-DFc electron density (before refinement). Residues I27, I337, H338 and V341 (cyan sticks) interact with fusidic acid.

The model containing three fusidic acid molecules at each of the three protomers revealed interactions of fusidic acid with residues I27 on TM1 and K334, I337, H338 and V341 on TM2 (Supplementary Figs 3 and 4a). Distances between ligand and amino acid side chains varied between 2.8 and 4.0 Å. These interactions are mainly of hydrophobic nature, with the exception of a hydrogen bond (2.8–3.2 Å distance) between His338 and the carboxylate moiety of fusidic acid (Supplementary Fig. 4a). The latter interaction is of special interest, as Poisson–Boltzmann calculations predicted different protonation probabilities for His338 in the L (50% probability), T (deprotonated) and O (protonated) state^10^. Moreover, structural and molecular simulation data indicated a large water accessibility for this TM1/TM2 groove His338/Glu346 region (Fig. 2), most likely to be induced by the L to T transition on ligand binding to the deep binding pocket in the periplasmic porter domain (Fig. 1)^10^. As His338 is predicted to be deprotonated in the T state, we assume that the carboxyl acid moiety of fusidic acid is likewise neutralized by protonation, and that the interaction with His338 is by hydrogen bonding only. The weaker electron densities found for the putative fusidic acid molecules localized at the same position at the L and O protomers compared with the densities observed for fusidic acid at the T protomer might reflect lower binding affinity due to the different protonation states of the His338 at each protomer^10^.

**Figure 3:**
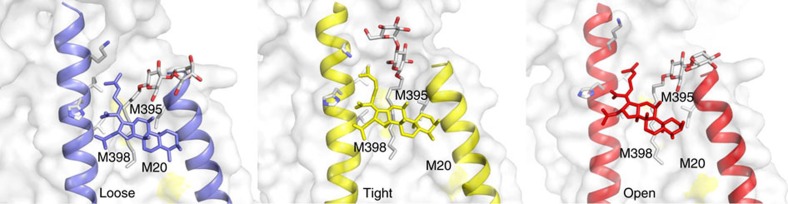
Localization of fusidic acid and DDM in each of the conformers of asymmetric AcrB. DDM molecules (white stick representation, O atoms are red) are bound to the TM1/TM2 groove distal to fusidic acid in the L (loose, blue), T (tight, yellow) and O (open, red) conformers of asymmetric AcrB. The sulfur atom positions of M20, M395 and M398 are indicated yellow in the white surface representation of the TM1/TM2 area of the AcrB protomers. Residues interacting with fusidic acid are represented as grey sticks.

We also observed clear additional electron densities in each protomer between TM1 and TM2 next to the fusidic acid molecule. We assigned these densities to dodecyl-β-D-maltoside (DDM; Fig. 3 and Supplementary Figs 4b and 5). Of note, in the previous published structures^14^ (pdb entries 4DX5 and 4DX7), DDM was assigned to this position as well (in the L and T protomers).

In the distal part of the TM1/TM2 groove, towards the middle of the lipid bilayer, the DDM alkyl chain is located at 6–9 Å distance to three methionine side chains (M20 on TM1, M395 and M398 on TM2; Fig. 3). Lipid alkyl chain interactions in TM regions of membrane proteins are often mediated by methionine residues^28^, which might indicate that *in vivo*, a lipid molecule (with two longer alkyl chains) occupies the TM1/TM2 groove of AcrB. In addition to the side chain interactions, the assigned DDM molecules appear to interact with fusidic acid via their alkyl chains (Supplementary Fig. 4b). Interestingly, the orientation of the maltoside headgroup in the T state is markedly different as that in the L and O states. Whereas in the latter two states the maltose moiety shows a single interaction with the L28 carbonyl oxygen, the headgroup is clamped to the protein by multiple hydrogen bonding interaction with PC1 subdomain-derived Asn298 and Asp301, and TM2 located Lys334 in the T state (Supplementary Fig. 4b). The alignment of the DDM appears to intensify its interaction with the dimethylallyl group of fusidic acid and might be an explanation for the more defined electron densities, which we assigned to fusidic acid in the T state. We speculate that *in vivo*, additional lipid-mediated hydrophobic interaction might stabilize the interaction between fusidic acid and AcrB inside the TM domain. *In vitro*, however, the exchange of lipid by detergent results in a possible detergent-mediated hydrophobic interaction as seen in the current complex structure.

The observed binding of fusidic acid to each of the three protomers is markedly different from earlier reported co-crystal structures^29^,^30^,^31^,^32^. These previous structures (at 3.5–3.8 Å resolution) represent the symmetric conformation of AcrB (that is, LLL conformation) and indicated binding of rhodamine 6G, ethidium, dequalinium, ciprofloxacin^29^, ampicillin^30^ or linezolid^31^ molecules in the central cavity comprised by the TM domain of each protomer (Supplementary Fig. 6).

In comparison, the protomers of the asymmetric AcrB structure at high resolution (2.5 Å), reported here, bind fusidic acid at the TM1/TM2 groove, outside the central cavity.

### I337 is important for fusidic acid and β-lactam resistance

To correlate the observation of fusidic acid binding to TM2 with the role of this binding site in efflux function, we set out to substitute the main interaction sites I337, H338 and V341 with Ala (and in addition H338 to Arg).

**Figure 4:**
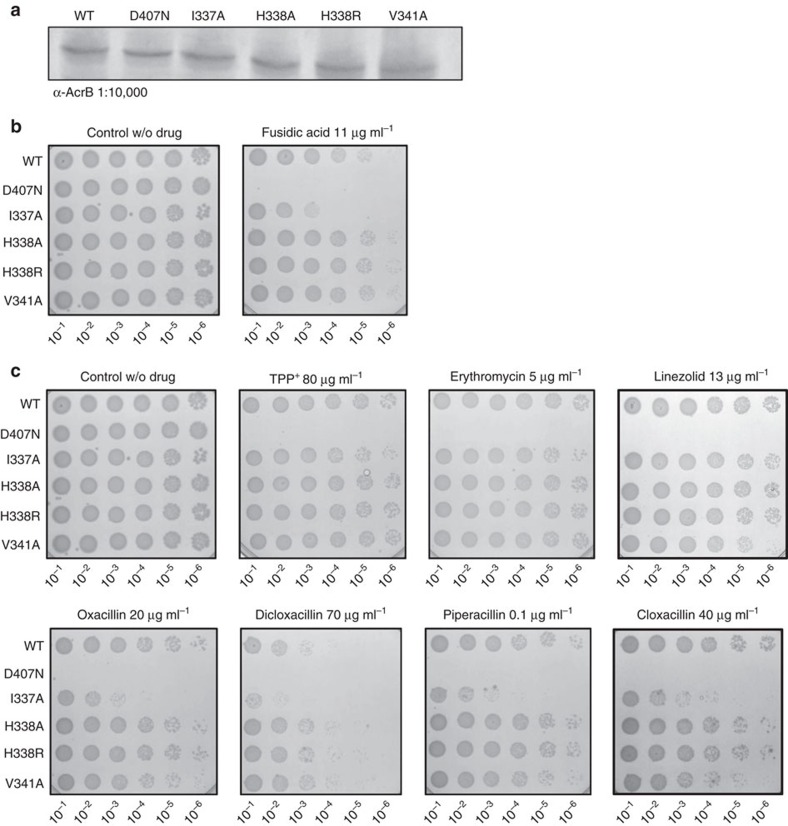
Expression and phenotypic analysis of AcrB wild-type and TM2 substitution variants on drug agar plates. (**a**) Western blot analysis of equal amounts of *E. coli* cells harbouring the indicated AcrB variants using rabbit α-AcrB. Plasmid born wild-type AcrB, D407N, I337A, H338A, H338R and V341A variants are produced to comparable levels in *E. coli* BW25113ΔacrB cells. (**b**,**c**) Drug LB agar plate dilution test with the indicated AcrB variants. *E. coli* BW25113ΔacrB was complemented with *acrB* wild-type or mutant genes expressed from plasmid. For the assay, colonies of these cells were picked and grown overnight in LB_Kan_ medium at 37 °C, diluted to OD_600_ 10^−1^–10^−6^ and 4 μl were spotted on an LB_Kan_ agar plate supplemented with (**b**) 11 μg ml^−1^ fusidic acid or (**c**) 80 μg ml^−1^ TPP^+^, 5 μg ml^−1^ erythromycin, 13 μg ml^−1^ linezolid, 20 μg ml^−1^ oxacillin, 70 μg ml^−1^ dicloxacillin, 0.1 μg ml^−1^ piperacillin, 40 μg ml^−1^ cloxacillin. Comparison between wild-type and the D407N mutant (deficient in proton translocation) clearly shows variant I337A to be more susceptible when exposed to fusidic acid or β-lactams, whereas in the presence of erythromycin, TPP^+^ or linezolid no difference in resistance was observed. Cell dilutions are indicated at the bottom of each plate.

The D407N, I337A, H338A, H338R and V341A substitution variants were equally well-expressed compared with wild-type AcrB from the same expression vector (Fig. 4a). We conducted fusidic acid susceptibility assay with *E. coli* BW25133*ΔacrB* cells harbouring either the wild-type *acrB* gene, the inactive variant *acrB_D407N* gene^9^,^33^ or one of the four substitution mutants using solid growth lysogeny broth (LB) supplemented with fusidic acid (11 μg ml^−1^). Although substitution of H338 and V341 show no apparent influence on the susceptibility of *E. coli* in the presence of fusidic acid, a pronounced effect was observed with *E. coli* containing the AcrB I337A substitution variant (Fig. 4b). Apparently, substitution of H338 with Ala or Arg was without severe consequence on fusidic acid or β-lactam efflux activity, whereas I337 was most sensitive to replacement for these substrates.

Analysis of growth of the substitution variants (Supplementary Fig. 7) on LB liquid medium containing fusidic acid (60 μg ml^−1^) exhibited inhibited growth of all variants compared with *E. coli* wild-type containing AcrB. The most severe growth inhibition was observed with the D407N substitution, as expected. From the putative TM2-binding site substitution variants, the I337A variant showed most severe growth inhibition. The severity of growth inhibition for all variants was D407N>I337A>H338R>V341A>H338A>wild-type. On basis of the localization of bound fusidic acid and its interaction with His338, we initially hypothesized that drugs with carboxylic acid moieties might be most affected by substitutions in the TM2, especially the H338 to Ala or Arg substitutions. We therefore included different β-lactam antibiotics (oxacillin, cloxacillin, dicloxacillin and piperacillin) as well as erythromycin, TPP^+^, linezolid, chloramphenicol, novobiocin, taurocholate, deoxycholate and rhodamine 6G (as controls) in the resistance assay on drug agar plates (Fig. 4c and Supplementary Fig. 8). Again, to our surprise, the I337A substitution variant was most growth inhibited of all substitution variants in the presence of drugs with carboxylic acid moieties, including all four β-lactam substrates, whereas no inhibiting effect could be observed compared with wild-type AcrB in the presence of the other tested substrates (Fig. 4c and Supplementary Fig. 8). The other putative TM2-binding site substitution variants showed little (for example, for V341A in the presence of oxacillin, cloxacillin, or piperacillin) or no growth inhibition in the presence of any of the substrates tested, with the exception of the growth inhibition in the presence of chloramphenicol for the V341A substitution variant (Supplementary Fig. 8).

### TM1–TM2 site protection by dicloxacillin and fusidic acid

**Figure 5:**
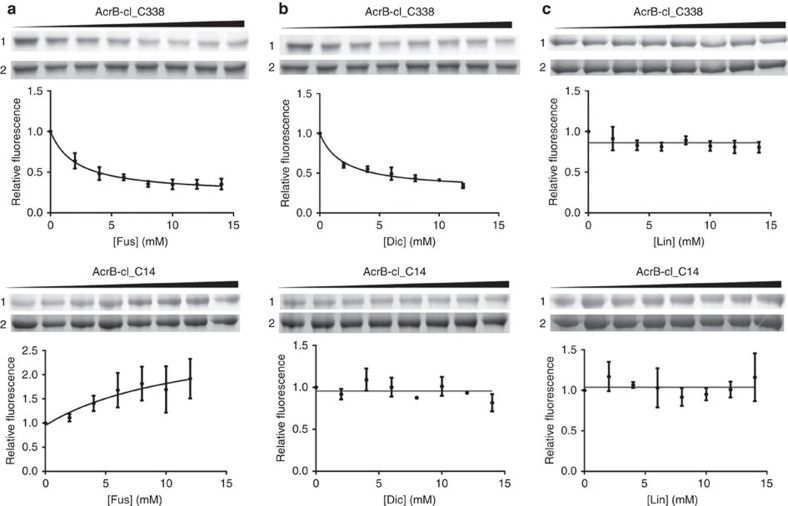
Substrate protection of MTS-rhodamine cross-linking for AcrB variants H338C and V14C. Protection by fusidic acid (**a**), dicloxacillin (**b**) or linezolid (**c**) against MTS-rhodamine modification of C338 at the TM1/2 groove or C14, located at TM1 near the cytoplasmic side (that is, remote from the TM1/2 groove). Membranes containing AcrB-cl_C338 or AcrB-cl_C14 in a cysteine-less (cl) background were incubated with the indicated concentration of fusidic acid (Fus), dicloxacillin (Dic) or linezolid (Lin) before MTS-rhodamine modification. For AcrB-cl_C338, the fluorescence signal of MTS-rhodamine-labelled protein reduces as the concentration of fusidic acid or dicloxacillin increases, indicating drug protection at the TM1/2 groove. For linezolid, no reduction was observed in dependence of the concentration, indicating the lack of protection. MTS-rhodamine modification of AcrB-cl_C14 in dependence of fusidic acid, linezolid or dicloxacillin showed no reduction of the labelling in dependence of the concentration; however, for fusidic acid, an increase of the MTS-rhodamine labelling was observed (see main text). Upper (AcrB-cl_C338) and lower (AcrB-cl_C14) panels, lane 1: in-gel fluorescence of MTS-rhodamine modified AcrB-cl_C338 or AcrB-cl_C14 in dependence of the drug concentration (from left to right increasing concentration of drug). Lane 2: the same SDS–PAA gel as shown in the upper lane stained with Coomassie indicated equal amount of AcrB-cl_C338 or AcrB-cl_C14 applied on the gel. The fluorescence signal from MTS-rhodamine modified AcrB-cl_C338 and AcrB-cl_C14 was quantified by the ImageQuant TL software and indicated as relative fluorescence with the signal in the absence of drug set to 1. The curves represent a nonlinear regression fit to a one-site saturation binding function. All the experiments were repeated at least three times (technical replicates) and the error bars represent s.e.m.

To verify the observed binding of fusidic acid to the putative TM1/TM2-binding site, we conducted cross-link protection experiments with a single-Cys AcrB variant (H338C). MTS-rhodamine labelling was done in membrane vesicles containing the AcrB-cl_C338 variant from which it was subsequently purified and subjected to SDS–PAGE and in-gel fluorescence analysis. Ninety percent of AcrB_C338 was modified by MTS-rhodamine (20 μM) within 15 min of incubation of the membranes on ice (Supplementary Fig. 9). Addition of fusidic acid or dicloxacillin to the membrane samples before addition of MTS-rhodamine resulted in a pronounced reduction of fluorescence signal of MTS-rhodamine modified AcrB_C338 in a concentration-dependent manner (Fig. 5a,b). In contrast, addition of linezolid did not have a protective effect against MTS-rhodamine labelling (Fig. 5c). As a control, we used the single cysteine variant V14C, with the modified residue located at the N-terminal part of TM1 near the cytoplasmic side of the TM domain, expected not to play a role in the binding or transport of drugs. MTS-rhodamine reacted with C14 at a similar rate compared with the modification of C338. The presence of dicloxacillin or linezolid at concentrations up to 15 mM did not change the extent of labelling with MTS-rhodamine (Fig. 5b,c). A slight increase of MTS-rhodamine labelling was observed in dependence of the increasing fusidic acid concentration (Fig. 5a). This effect is possibly due to the perturbing effect of fusidic acid to the AcrB-containing membrane, slightly facilitating the reaction of MTS-rhodamine with the thiol group of the V14C variant. Importantly, the effect is the exact opposite of the observation with the H338C variant, where the presence of fusidic acid is protecting against labelling with MTS-rhodamine. If the perturbing effect also would facilitate labelling of C338, then the observed protective effect of fusidic acid against MTS-rhodamine cross-linking is even more remarkable (Fig. 5a). The calculated apparent *K*_i_ (inhibition constant of MTS-rhodamine labelling) for fusidic acid and dicloxacillin is ∼2.1 and 2.0 mM, respectively. These results revealed that fusidic acid and dicloxacillin are able to bind at the TM1/2 groove and protect the cysteine at position 338 for modification with MTS-rhodamine.

Carboxylate drugs are considered to be negatively charged at neutral pH (p*K*_a_ values: oxacillin: 2.7, piperacillin: 3.5, dicloxacillin: 3.75 and fusidic acid: 4.7–5.3); however, due to their hydrophobic moieties, these compounds immerse into the outer leaflet of the inner membrane on entry (from the external medium) into the periplasmic space. Near the membrane surface, the pH is expected to be lower than the bulk pH due to accumulation of surface-associated protons^34^, shifting the equilibrium towards the protonation of the carboxylic acid moieties and immersion of the drug into the outer leaflet of the inner membrane. Based on the previously determined protonation state of H338 in the T state^10^, we presume H338 to be deprotonated, whereas the carboxylate ligand is protonated. If so, interaction of fusidic acid, oxacillin, piperacillin or dicloxacillin with H338 is hydrogen bonding-mediated rather than by electrostatic interaction and might explain the lack of a severe effect on efflux activity in the H338 to Ala or Arg substitution variant (Fig. 4c). The key factor for carboxylate drug binding and transport appears to be I337, most probably establishing hydrophobic interactions with fusidic acid or the β-lactams oxacillin, cloxacillin, dicloxacillin and piperacillin. Possibly, the DDM molecule observed in the structure (and speculatively a lipid alkyl chain *in vivo*) might stabilize substrate binding as well. Despite the rather hydrophobic nature of substrate binding to the TM1/TM2 area, the common denominator of those substrates tested and affected by the I337A substitution is the presence of a carboxylic acid moiety. As the interaction of the carboxyl group of the substrates with H338 does not appear to be supercritical, later stages in the transport pathway, apparently involving I337, might cause the substrate-specific phenotype.

Based on the observed differences in the densities and anomalous signal of 24-bromofusidic acid and the number of different side chain interactions with the ligand in the three asymmetric protomers (Supplementary Fig. 4a), we speculate that the drug binds most tightly in the T conformation.

## Discussion

**Figure 6:**
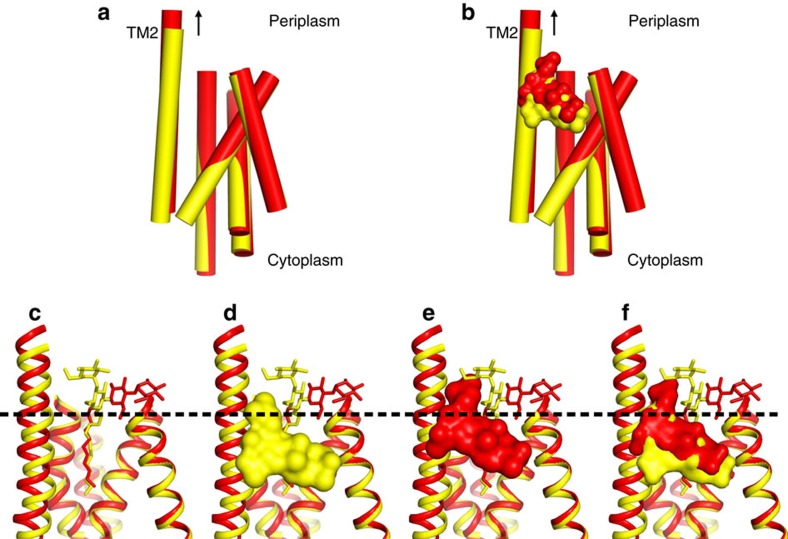
Congruent movement of TM2 and fusidic acid in the transition from T to O. (**a**,**b**) Side view parallel to the membrane plane of superimposed five-TM repeat 1 (TM1+TM3–6) in the T and O state (yellow and red, respectively). (**a**) TM2 moves upward towards the periplasm by ∼2.5 Å in the transition from T to O. (**b**) The upward movement of TM2 relocates bound fusidic acid towards the periplasmic side of the membrane (surface representation, yellow: position in the T conformation, red: position in the O conformation). (**c**) Localization of the membrane/periplasmic space interface based on the position of the alkyl chains and hydrophilic maltose moieties of the DDM molecules co-crystallized with AcrB (dashed line). (**d**) Localization of fusidic acid molecule in complex with AcrB in the T conformer. (**e**) Localization of fusidic acid molecule in complex with AcrB in the O conformer. (**f**) Superimposition of the T and O conformers including the fusidic acid and DDM molecules.

Previous structural analysis led to the hypothesis that the pseudosymmetric five-TM repeats of the AcrB TM domain undergo a lateral shear motion on protonation of the titratable D407 and D408 residues^10^. This conformational change also leads to a concomitant coil-to-helix transition in TM8, as well as TM2 helix translation towards the periplasm^10^. Indeed, superimposition of the AcrB T and O conformers indicates an upward movement of TM2 during the T to O transition of ∼2.5 Å (Fig. 6a,b). In accordance, the bound fusidic acid molecule (Fig. 6d–f) likewise shifts 2.5 Å towards the periplasmic side. This shift causes the movement of fusidic acid from the hydrophobic core of the outer leaflet of the inner membrane to the more hydrophilic headgroup area of the phospholipid membrane/periplasmic space interface. Thus, on T to O transition, due to an upward ‘helix shift' of TM2, the drug is dragged in part out of the membrane towards the periplasm (Fig. 6). A large part of the fusidic acid alkyl chain is also displaced during this T to O transition from the membrane towards the AcrB periplasmic domain. In the O conformation, where TM2 is in the ‘up' position, fusidic acid locates slightly below a small hydrophobic cave made up of L300, V32, V333, I337 and the alkyl chain of K334 (Supplementary Fig. 10).

Here, drug-binding affinity might change due to the concurrent movement of TM2 and fusidic acid to a different environment and leads to the release of fusidic acid at the periplasmic side. Once TM2 returns to its down state in the O to L transition, due to the relative motion of the two five-TM repeats of the AcrB TM domain caused by deprotonation of the catalytic D407 and D408 residues^10^, the binding site is partially re-established and might function as a low-affinity binding site in the L protomer, which on transition to the T state turns into a higher affinity site (Supplementary Fig. 4a).

Further transport of fusidic acid towards the periplasmic binding pockets is difficult to predict on basis of current knowledge. One possibility would be the recently calculated ‘PC1/PN2 down' pathway^26^, describing movement of drugs from above the TM1/TM2 groove towards the deep binding pocket. The entrance of this pathway would be pseudosymmetric to the TM7/TM8 groove entrance as part of the vestibule pathway^14^,^18^,^25^.

**Figure 7:**
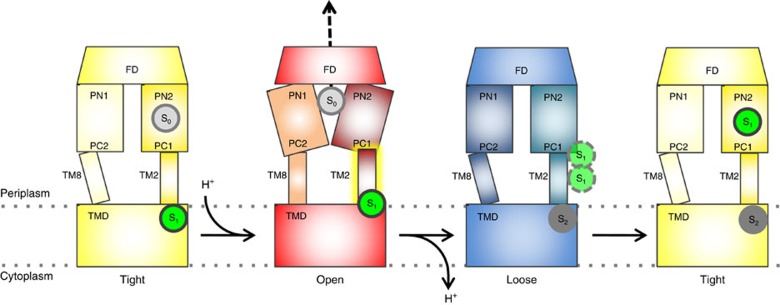
Helix shift mechanism for carboxylate drug transport from the membrane to the deep binding pocket. A carboxylate drug substrate (S_1_ in green circle) is associated with TM2 in the T state (left, yellow, Tight). Protonation events at the D407/D408 residues in the transmembrane domain (TMD) drive the transition from T to O (red, Open). This causes TM2 to move upwards perpendicular to the membrane plane, by ∼2.5 Å. The carboxylate substrate (S_1_) is shifted upwards along with TM2 and reaches the periplasmic space/membrane interface. Subsequently, substrate is released from the membrane, a process that might be facilitated by a hydrophobic cavity just above the membrane plane (red, Open, Supplementary Fig. 10). In the L state, the released substrate can possibly utilize the recently described ‘PN2/PC1 down' pathway^26^ from the membrane proximal part of PN2/PC1 into the deep binding pocket (right, yellow, Tight). On T to O transition, the newly captured carboxylate drug is transported via the funnel structure of the AcrB funnel domain (FD) towards TolC. After substrate release from the open state, new substrate can be captured in the loose state (dark grey, S_2_).

As is summarized in Fig. 7, carboxylate drugs are postulated to be elevated from the membrane during the T to O transition by means of the TM2 helix upward shift, further transported during the O to L transition via the PC1/PN2 down pathway and bound to the deep binding pocket in the L to T transition. This mechanism is linked directly to the protonation and deprotonation events during the T to O and O to L transitions, and mediated by the up and downward movements of TM2, respectively.

The results presented here give for the first time a direct indication that AcrB drug binding sites can be specific for drugs with defined properties, in this case for carboxylated drugs or drugs containing putative negatively charged moieties.

## Methods

### Bacterial strains and growth conditions

*E. coli* MachT1 (Life Technologies) cells were used as host for cloning procedures. *E. coli* C43 (DE3)*ΔacrAB*^35^ harbouring pET24acrB_His_^36^ was used for protein overproduction. LB medium and LB agar were used for routine bacterial growth at 37 °C. Kanamycin (Applichem) was used at 50 μg ml^−1^ (Kan^50^).

### Cloning of acrB gene

The *acrB* gene was amplified from chromosomal *E. coli* DNA using acrBfor and acrBrev primers^36^ (Supplementary Table 2). The amplified *acrB* gene was cloned into pET24a (Novagen) via NdeI and XhoI restriction sites. The resulting plasmid pET24acrB_His_ was verified by sequencing (Eurofins).

### Synthesis of 24-bromofusidic acid

Bromofusidic acid was custom-synthesized by Basilea Pharmaceutica AG (Basel, Switzerland) and was prepared from fusidic acid (CAS 6990-06-3, Sigma-Aldrich) following a five-step procedure reported in patent WO2005/007669A1. The final product was validated by HPLC, liquid cromatography–mass spectrometry and ^1^H-NMR methods (Supplementary Methods).

### Site-directed mutagenesis

pET24acrB_His_^36^ served as template for site-directed mutagenesis. Amino acid substitution was achieved using the ExSite protocol (Stratagene) with 5'-phosphorylated primers (Supplementary Table 2). Mutations were verified by sequencing (Eurofins).

### DARPin protein production and purification

Briefly, *E. coli* XL1-Blue cells harbouring pQE30-DARPin^18^ were grown overnight in LB liquid medium supplemented with 50 μg ml^−1^ kanamycin (LB_Kan_) at 37 °C. Overnight cultures were inoculated into fresh LB_Kan_ liquid medium and induction was performed with 0.5 mM isopropyl-β-D-thiogalactoside (final concentration) at OD_600_ of 0.7. Induced cultures were grown overnight at 37 °C. Cells were harvested by centrifugation and suspended in Buffer A (50 mM Tris pH 7.5, 400 mM NaCl and 10 mM Imidazol). Cells were disrupted by triple passage through a cell disruptor (Constant System, Inc., Northants, UK). Insoluble material was removed by centrifugation at 160,000 *g* for 1 h. Supernatant was added to Ni-NTA agarose beads (30210, Qiagen) and incubated for 1 h with rotation. Subsequently, Ni-NTA agarose beads were washed with 30 and subsequently 20 column volumes of Buffer B (50 mM Tris pH 7.5, 400 mM NaCl, 20 mM Imidazol, 10% Glycerol) and Buffer C (50 mM Tris pH 7.5, 400 mM NaCl, 50 mM Imidazol and 10% Glycerol), respectively. DARPin proteins were eluted with Buffer D (50 mM Tris pH 7.5, 400 mM NaCl, 250 mM Imidazol and 10% Glycerol).

### AcrB protein production and purification

*E. coli* C43 (DE3) Δ*acrAB* harbouring pET24acrB_His_^17^,^36^ was grown overnight in LB_Kan_ liquid medium at 37 °C. Overnight cultures were inoculated into fresh LB_Kan_ liquid medium, grown till OD_600_ of 0.8 before induction with 0.5 mM isopropyl-β-D-thiogalactoside (final concentration) and allowed to grow at 20 °C for another 16 h. Cells were harvested by centrifugation and suspended in Buffer A (20 mM Tris pH 8.0, 500 mM NaCl, 2 mM MgCl_2_ and 0.2 mM diisopropyl fluorophosphate). Cells were disrupted by triple passage through a cell disruptor (Constant System Inc.). Insoluble material was removed by centrifugation at 23,000 *g* for 15 min. Cell membranes were collected by centrifugation at 160,000 *g* for 1 h. Subsequently, membranes were suspended in Buffer B (20 mM Tris pH 7.5, 150 mM NaCl, 10 mM Imidazol and 10% Glycerol) and solubilized with 1% dodecyl maltoside (D-97002-C, DDM, Glycon, final concentration) at 4 °C for 1 h. Solubilized membranes were centrifuged at 160,000 g for 30 min. The supernatant was mixed with Ni-NTA agarose beads (30210, Qiagen) and incubated for 1 h with rotation. Subsequently, Ni-NTA agarose beads were washed with 40 and subsequently with 30 column volumes of Buffer B (20 mM Tris pH 7.5, 150 mM NaCl, 20 mM Imidazol, 10% Glycerol and 0.03% DDM) and Buffer C (20 mM Tris pH 7.5, 150 mM NaCl, 40 mM Imidazol, 10% Glycerol and 0.03% DDM), respectively. AcrB protein was eluted with Buffer D (20 mM Tris pH 7.5, 150 mM NaCl, 220 mM Imidazol, 10% Glycerol and 0.03% DDM).

### Drug agar plate assay

Drug agar plate assays were performed as previously described^44^. In short, colonies of *E. coli* BW25113 Δ*acrB* harbouring pET24acrB_His_ wild-type and various mutants were picked and grown overnight in LB_Kan_ medium at 37 °C. Dilution of the cultures to OD_600_ 10^−1^–10^−6^ were prepared and 4 μl were spotted on an LB_Kan_ agar plate supplemented with drug (fusidic acid: 11 μg ml^−1^, TPP^+^: 80 mg ml^−1^, erythromycin: 5 μg ml^−1^, oxacillin: 20 μg ml^−1^, cloxacillin: 40 μg ml^−1^, dicloxacillin: 70 μg ml^−1^, piperacillin: 0.1 μg ml^−1^, linezolid: 13 μg ml^−1^, taurocholate: 6 μg ml^−1^, deoxycholate: 5 μg ml^−1^, rhodamine 6G: 25 μg ml^−1^, chloramphenicol: 0.75 μg ml^−1^, novobiocin: 8 μg ml^−1^). Plates were incubated overnight at 37 °C.

### Western blot analysis

Whole-cell proteins extracted from *E. coli* BW25113 Δ*acrB* harbouring pET24acrB_His_ wild-type and various variants were resolved by 12.5% SDS–PAGE gels and transferred onto nitrocellullose membrane. The membrane was incubated with anti-AcrB antibody (dilution of 1:10,000; Neosystems, France, custom-antibody) for 90 min. Subsequently, the blot was treated with anti-rabbit IgG (whole molecule)-alkaline phosphatase antibody (dilution of 1:1,500; A3687, Sigma-Aldrich, St. Louis, USA) for 90 min. NBT (nitro-blue tetrazolium chloride) and BCIP (5-bromo-4-chloro-3'-indolyphosphate p-toluidine salt) were used for blot development. Unmodified western blotting of Fig. 4a is shown in Supplementary Fig. 11

### Growth assay

Colonies of *E. coli* BW25113Δ*acrB* harbouring pET24acrB_His_ wild-type and various variants were selected and grown in LB_Kan_ at 37 °C for 6 h. Cultures were diluted to OD_600_ of 0.02 and 50 μl was used to inoculate 100 μl of LB_Kan_ supplemented with fusidic acid at different concentrations in a 96-well microtitre plate as indicated. The microtitre plate was placed at 37 °C and the OD_600_ was measured using a Tecan Infinite M200 Reader every 20 min for a period of 11 h.

### Substrate protection cross-linking assay

Membranes were prepared as shown above. A membrane aliquot (∼0.1 g) was incubated with 5 mM dithiothreitol for 30 min on ice, followed by two washing steps with ice-cold cross-linking buffer (CLB: 20 mM Tris/Cl pH 7.0 and 0.5 M NaCl) and collection by ultracentrifugation at 137,000 *g* for 30 min. A total of 5 mg membranes was suspended in 150 μl CLB containing 0–14 mM fusidic acid or other substrates as indicated, and sonicated briefly (amplitude=10%, 2 × 1 s, Sonoplus, Bandelin Electronic, Berlin, Germany), before incubation at 18 °C for 16 h. Subsequently, MTS-rhodamine (20 μM final concentration) was added and the sample was incubated for 15 min on ice. The reaction was stopped by addition of methoxypolyethylene glycol maleimide (2 mM final concentration). Membranes were subsequently washed twice with ice-cold CLB (137,000 g for 20 min), and suspended in CLB containing 1% SDS and 2 mM methoxypolyethylene glycol maleimide before purification of AcrB with HisTrap HP beads on a spin column. Purified AcrB was subjected to SDS–PAGE after which the fluorescence signal was detected on a ImageQuant LAS 4000 (Excitation with Epi-Green (Cy3) and emission filter of 575DF20 (Cy3)) (GE Healthcare BioSciences AB, Uppsala, Sweden) before staining with Coomassie Brilliant Blue. Uncropped images of in-gel fluorescence and Coomassie-stained SDS–PAA gels of Fig. 5 (Lane 1 and Lane 2) are shown in Supplementary Fig. 12 (AcrB-cl_C338) and Supplementary Fig. 13 (AcrB-cl_C14).

### Crystallization of AcrB–DARPin–fusidic acid complex

For crystallization purposes, AcrB was mixed with DARPin in a 1:2 molar ratio and incubated on ice for 10 min. Excess DARPin was removed by size-exclusion chromatography (Superose 6, GE Healthcare) with buffer containing 20 mM Tris pH 7.5, 150 mM NaCl and 0.03% DDM. AcrB/DARPin was concentrated and used for hanging drop crystallization at 10–15 mg ml^−1^. Fusidic acid (4 mM) was added before crystallization. The reservoir contained 50 mM ADA pH 6.9, 5% Glycerol, 5–10% PEG4000 and 150–250 mM ammonium sulfate. Crystals appeared in 1 week and grew to their optimum size (100 × 70 × 200 μm) within additional 1–2 weeks. AcrB/DARPin/24-bromofusidic acid crystals were obtained under the same conditions as described above, with the omission of fusidic acid before soaking the apo-AcrB/DARPin crystals with 8 mM brominated fusidic acid for 5 days.

### X-ray diffraction data set analysis and refinement procedure

A data set of P2_1_2_1_2_1_ crystals was obtained at beamline X06DA of the Swiss Light Source (Paul Scherrer Institute). Data reduction was done with the XDS package^37^. Structures were refined using phenix.refine from the PHENIX package^27^ or using REFMAC5 (ref. 38) and validated with MolProbity^39^. Model rebuilding was done using COOT^40^. Over 99% of residues are in favoured regions of the Ramachandran plot and only 0.2% of the residues are outliers. A stereo image of part of the electron 2*F*_o_−*F*_c_ density is shown in Supplementary Fig. 14. For superimpositions, the programme SUPERPOSE^41^ was used. Figures were created using PyMOL (Schrödinger, LLC). AcrB crystals with brominated fusidic acid bound were measured at 0.9187 Å at beamline BM14 (European Synchrotron Radiation Facility, Grenoble, France). Data sets were processed and scaled with the XDS package, the unmerged data was set up with ShelxC^42^ and the anomalous signal of bromine was located by ANODE^43^.

### Data availability

The atomic coordinates and structure factors of the wild-type AcrB in complex with DARPins and fusidic acid have been deposited at the Protein Data Bank with accession number 5JMN. The authors declare that all other data supporting the findings of this study are available within the paper and its Supplementary Information files or are available from the corresponding author upon request.

## Additional information

**How to cite this article:** Oswald, C. *et al*. Transport of lipophilic carboxylates is mediated by transmembrane helix 2 in multidrug transporter AcrB. *Nat. Commun.* **7,** 13819 doi: 10.1038/ncomms13819 (2016).

**Publisher's note:** Springer Nature remains neutral with regard to jurisdictional claims in published maps and institutional affiliations.

## Supplementary Material

Supplementary Information Supplementary Figures, Supplementary Tables and Supplementary Methods.

## References

[b1] Bush K. . Tackling antibiotic resistance. Nat. Rev. Microbiol. 9, 894–896 (2011). 22048738 10.1038/nrmicro2693 PMC4206945

[b2] Viveiros M. . Antibiotic stress, genetic response and altered permeability of *E. coli*. PLoS ONE 2, e365 (2007). 17426813 10.1371/journal.pone.0000365 PMC1838523

[b3] Piddock L. J. Clinically relevant chromosomally encoded multidrug resistance efflux pumps in bacteria. Clin. Microbiol. Rev. 19, 382–402 (2006). 16614254 10.1128/CMR.19.2.382-402.2006 PMC1471989

[b4] Nikaido H. Multidrug resistance in bacteria. Annu. Rev. Biochem. 78, 119–146 (2009). 19231985 10.1146/annurev.biochem.78.082907.145923 PMC2839888

[b5] Pos K. M. Drug transport mechanism of the AcrB efflux pump. Biochim. Biophys. Acta 1794, 782–793 (2009). 19166984 10.1016/j.bbapap.2008.12.015

[b6] Nikaido H. Multidrug efflux pumps of Gram-negative bacteria. J. Bacteriol. 178, 5853–5859 (1996). 8830678 10.1128/jb.178.20.5853-5859.1996 PMC178438

[b7] Guan L. & Nakae T. Identification of essential charged residues in transmembrane segments of the multidrug transporter MexB of *Pseudomonas aeruginosa*. J. Bacteriol. 183, 1734–1739 (2001). 11160105 10.1128/JB.183.5.1734-1739.2001 PMC95059

[b8] Takatsuka Y. & Nikaido H. Threonine-978 in the transmembrane segment of the multidrug efflux pump AcrB of *Escherichia coli* is crucial for drug transport as a probable component of the proton relay network. J. Bacteriol. 188, 7284–7289 (2006). 17015667 10.1128/JB.00683-06 PMC1636234

[b9] Seeger M. A., von Ballmoos C., Verrey F. & Pos K. M. Crucial role of Asp408 in the proton translocation pathway of multidrug transporter AcrB: evidence from site-directed mutagenesis and carbodiimide labeling. Biochemistry 48, 5801–5812 (2009). 19425588 10.1021/bi900446j

[b10] Eicher T. . Coupling of remote alternating-access transport mechanisms for protons and substrates in the multidrug efflux pump AcrB. Elife 3, e03145 (2014). 10.7554/eLife.03145 PMC4359379 25248080

[b11] Eda S., Maseda H. & Nakae T. An elegant means of self-protection in Gram-negative bacteria by recognizing and extruding xenobiotics from the periplasmic space. J. Biol. Chem. 278, 2085–2088 (2003). 12460990 10.1074/jbc.C200661200

[b12] Tikhonova E. B., Wang Q. & Zgurskaya H. I. Chimeric analysis of the multicomponent multidrug efflux transporters from gram-negative bacteria. J. Bacteriol. 184, 6499–6507 (2002). 12426337 10.1128/JB.184.23.6499-6507.2002 PMC135444

[b13] Elkins C. A. & Nikaido H. Substrate specificity of the RND-type multidrug efflux pumps AcrB and AcrD of *Escherichia coli* is determined predominantly by two large periplasmic loops. J. Bacteriol. 184, 6490–6498 (2002). 12426336 10.1128/JB.184.23.6490-6498.2002 PMC135441

[b14] Eicher T. . Transport of drugs by the multidrug transporter AcrB involves an access and a deep binding pocket that are separated by a switch-loop. Proc. Natl Acad. Sci. USA 109, 5687–5692 (2012). 22451937 10.1073/pnas.1114944109 PMC3326505

[b15] Nakashima R., Sakurai K., Yamasaki S., Nishino K. & Yamaguchi A. Structures of the multidrug exporter AcrB reveal a proximal multisite drug-binding pocket. Nature 480, 565–569 (2011). 22121023 10.1038/nature10641

[b16] Murakami S., Nakashima R., Yamashita E., Matsumoto T. & Yamaguchi A. Crystal structures of a multidrug transporter reveal a functionally rotating mechanism. Nature 443, 173–179 (2006). 16915237 10.1038/nature05076

[b17] Seeger M. A. . Structural asymmetry of AcrB trimer suggests a peristaltic pump mechanism. Science 313, 1295–1298 (2006). 16946072 10.1126/science.1131542

[b18] Sennhauser G. . Drug export pathway of multidrug exporter AcrB revealed by DARPin inhibitors. PLoS Biol. 5, e7 (2007). 17194213 10.1371/journal.pbio.0050007 PMC1717020

[b19] Nakashima R. . Structural basis for the inhibition of bacterial multidrug exporters. Nature 500, 102–106 (2013). 23812586 10.1038/nature12300

[b20] Sjuts H. . Molecular basis for inhibition of AcrB multidrug efflux pump by novel and powerful pyranopyridine derivatives. Proc. Natl Acad. Sci. USA 113, 3509–3514 (2016). 26976576 10.1073/pnas.1602472113 PMC4822567

[b21] Schuldiner S. EmrE, a model for studying evolution and mechanism of ion-coupled transporters. Biochim. Biophys. Acta 1794, 748–762 (2009). 19167526 10.1016/j.bbapap.2008.12.018

[b22] Fluman N., Adler J., Rotenberg S. A., Brown M. H. & Bibi E. Export of a single drug molecule in two transport cycles by a multidrug efflux pump. Nat. Commun. 5, 1–9 (2014). 10.1038/ncomms5615 25105370

[b23] Tal N. & Schuldiner S. A coordinated network of transporters with overlapping specificities provides a robust survival strategy. Proc. Natl Acad. Sci. USA 106, 9051–9056 (2009). 19451626 10.1073/pnas.0902400106 PMC2690002

[b24] Liu A. . Antibiotic sensitivity profiles determined with an *Escherichia coli* gene knockout collection: generating an antibiotic bar code. Antimicrob. Agents Chemother. 54, 1393–1403 (2010). 20065048 10.1128/AAC.00906-09 PMC2849384

[b25] Murakami S., Nakashima R., Yamashita E. & Yamaguchi A. Crystal structure of bacterial multidrug efflux transporter AcrB. Nature 419, 587–593 (2002). 12374972 10.1038/nature01050

[b26] Yao X.-Q., Kimura N., Murakami S. & Takada S. Drug uptake pathways of multidrug transporter AcrB studied by molecular simulations and site-directed mutagenesis experiments. J. Am. Chem. Soc. 135, 7474–7485 (2013). 23627437 10.1021/ja310548h

[b27] Adams P. D. . PHENIX: a comprehensive Python-based system for macromolecular structure solution. Acta Crystallogr. D Biol Crystallogr. 66, 213–221 (2010). 20124702 10.1107/S0907444909052925 PMC2815670

[b28] Brosnan J. T. & Brosnan M. E. The sulfur-containing amino acids: an overview. J. Nutr. 136, 1636S–1640S (2006). 16702333 10.1093/jn/136.6.1636S

[b29] Yu E. W. . Structural basis of multiple drug-binding capacity of the AcrB multidrug efflux pump. Science 300, 976–980 (2003). 12738864 10.1126/science.1083137

[b30] Tornroth-Horsefield S. . Crystal structure of AcrB in complex with a single transmembrane subunit reveals another twist. Structure 15, 1663–1673 (2007). 18073115 10.1016/j.str.2007.09.023

[b31] Hung L.-W. . Crystal structure of AcrB complexed with linezolid at 3.5 Å resolution. J. Struct. Funct. Genomics 14, 71–75 (2013). 23673416 10.1007/s10969-013-9154-x PMC3679416

[b32] Drew D. . The structure of the efflux pump AcrB in complex with bile acid. Mol. Membr. Biol. 25, 677–682 (2008). 19023693 10.1080/09687680802552257

[b33] Seeger M. A. . Engineered disulfide bonds support the functional rotation mechanism of multidrug efflux pump AcrB. Nat. Struct. Mol. Biol. 15, 199–205 (2008). 18223659 10.1038/nsmb.1379

[b34] Wolf M. G., Grubmüller H. & Groenhof G. Anomalous surface diffusion of protons on lipid membranes. Biophys. J. 107, 76–87 (2014). 24988343 10.1016/j.bpj.2014.04.062 PMC4119267

[b35] Miroux B. & Walker J. E. Over-production of proteins in *Escherichia coli*: mutant hosts that allow synthesis of some membrane proteins and globular proteins at high levels. J. Mol. Biol. 260, 289–298 (1996). 8757792 10.1006/jmbi.1996.0399

[b36] Pos K. M. & Diederichs K. Purification, crystallization and preliminary diffraction studies of AcrB, an inner-membrane multi-drug efflux protein. Acta Crystallogr. D Biol. Crystallogr. 58, 1865–1867 (2002). 12351840 10.1107/s0907444902013963

[b37] Kabsch W. XDS. Acta Crystallogr. D Biol. Crystallogr. 66, 125–132 (2010). 20124692 10.1107/S0907444909047337 PMC2815665

[b38] Murshudov G. N. . REFMAC5 for the refinement of macromolecular crystal structures. Acta Crystallogr. D Biol. Crystallogr. 67, 355–367 (2011). 21460454 10.1107/S0907444911001314 PMC3069751

[b39] Chen V. B. . *MolProbity *: all-atom structure validation for macromolecular crystallography. Acta Crystallogr. D Biol. Crystallogr. 66, 12–21 (2010). 20057044 10.1107/S0907444909042073 PMC2803126

[b40] Emsley P., Lohkamp B., Scott W. G. & Cowtan K. Features and development of Coot. Acta Crystallogr. D Biol. Crystallogr. 66, 486–501 (2010). 20383002 10.1107/S0907444910007493 PMC2852313

[b41] Krissinel E. & Henrick K. Secondary-structure matching (SSM), a new tool for fast protein structure alignment in three dimensions. Acta Crystallogr. D Biol. Crystallogr. 60, 2256–2268 (2004). 15572779 10.1107/S0907444904026460

[b42] Sheldrick G. M. Experimental phasing with SHELXC / D / E : combining chain tracing with density modification. Acta Crystallogr. D Biol. Crystallogr. 66, 479–485 (2010). 20383001 10.1107/S0907444909038360 PMC2852312

[b43] Thorn A. & Sheldrick G. M. ANODE: anomalous and heavy-atom density calculation. J. Appl. Crystallogr. 44, 1285–1287 (2011). 22477786 10.1107/S0021889811041768 PMC3246834

[b44] Adler J., Lewinson O. & Bibi E. Role of a conserved membrane-embedded acidic residue in the multidrug transporter MdfA. Biochemistry 43, 518–525 (2004). 14717607 10.1021/bi035485t

