# Supplementary material for: Transport of lipophilic carboxylates is mediated by transmembrane helix 2 in multidrug transporter AcrB
Source: Nat Commun. 2016 Dec 16;7:13819. doi: 10.1038/ncomms13819 (PMC5171871; doi:10.1038/ncomms13819)
Supplement: Supplementary Information — Supplementary Figures, Supplementary Tables and Supplementary Methods. [file ncomms13819-s1.pdf]

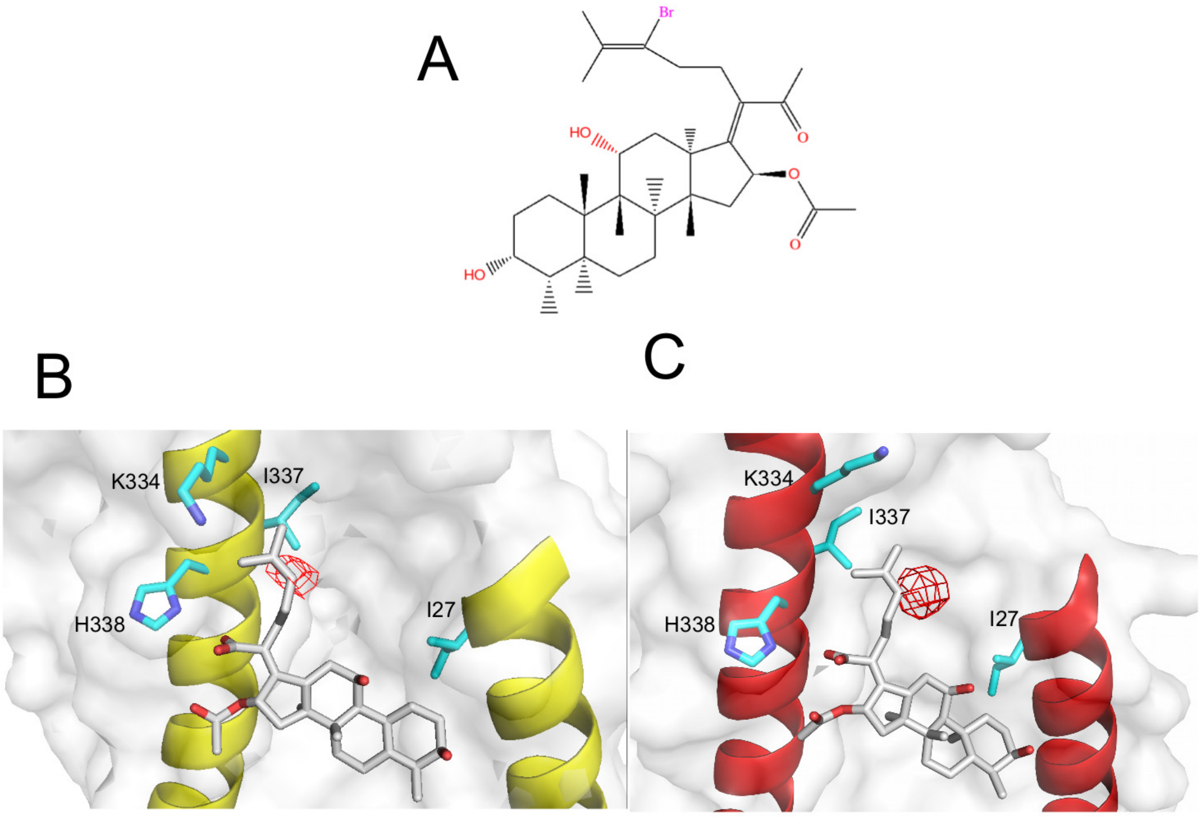

Supplementary Figure 1 - 24-Bromofusidic acid bound at TM1-TM2

A Chemical structure of 24-bromofusidic acid. **B, C** Anomalous density for the bromine atom at TM1/TM2 in **B** tight and **C** open monomer, contoured at 5 σ, 1.3098 e Å^3 (red mesh). The anomalous signal of the bromine atom is located at position C24.

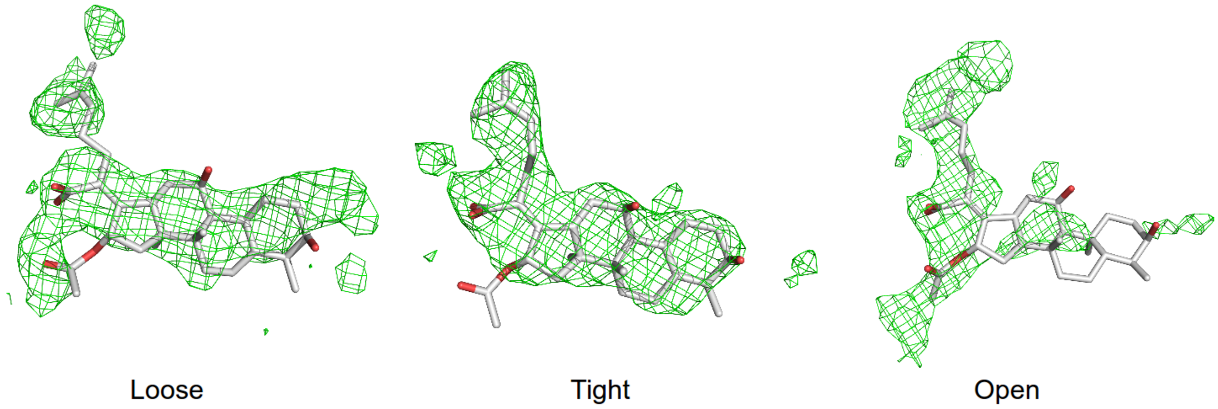

Supplementary Figure 2 - Polder and Fo-Fc electron density map for fusidic acid at TM2 groove

Polder maps contoured at 3.5, 4.0 and 3.0 σ for fusidic acid bound at loose, tight and open conformer, respectively. Polder maps were calculated by phenix.polder[27]. Fusidic acid is shown as sticks (carbon = white; oxygen = red).

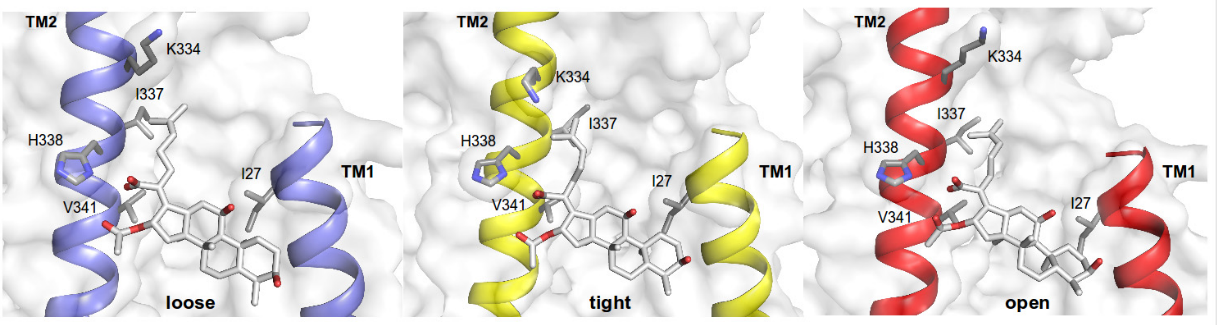

Supplementary Figure 3 - Localization of fusidic acid in each of the three protomers at the TM1/TM2 groove of asymmetric AcrB

Fusidic acid (white stick presentation with oxygen atoms in red) between TM1 (right helix) and TM2 (left helix) in the loose (on the left, blue), tight (middle, yellow), and open (right, red) conformer of asymmetric AcrB. On TM1, I27 is indicated as grey stick, on TM2, from top to bottom, K334, I337, H338 and V341 side chains are indicated in grey sticks (N atoms in blue).

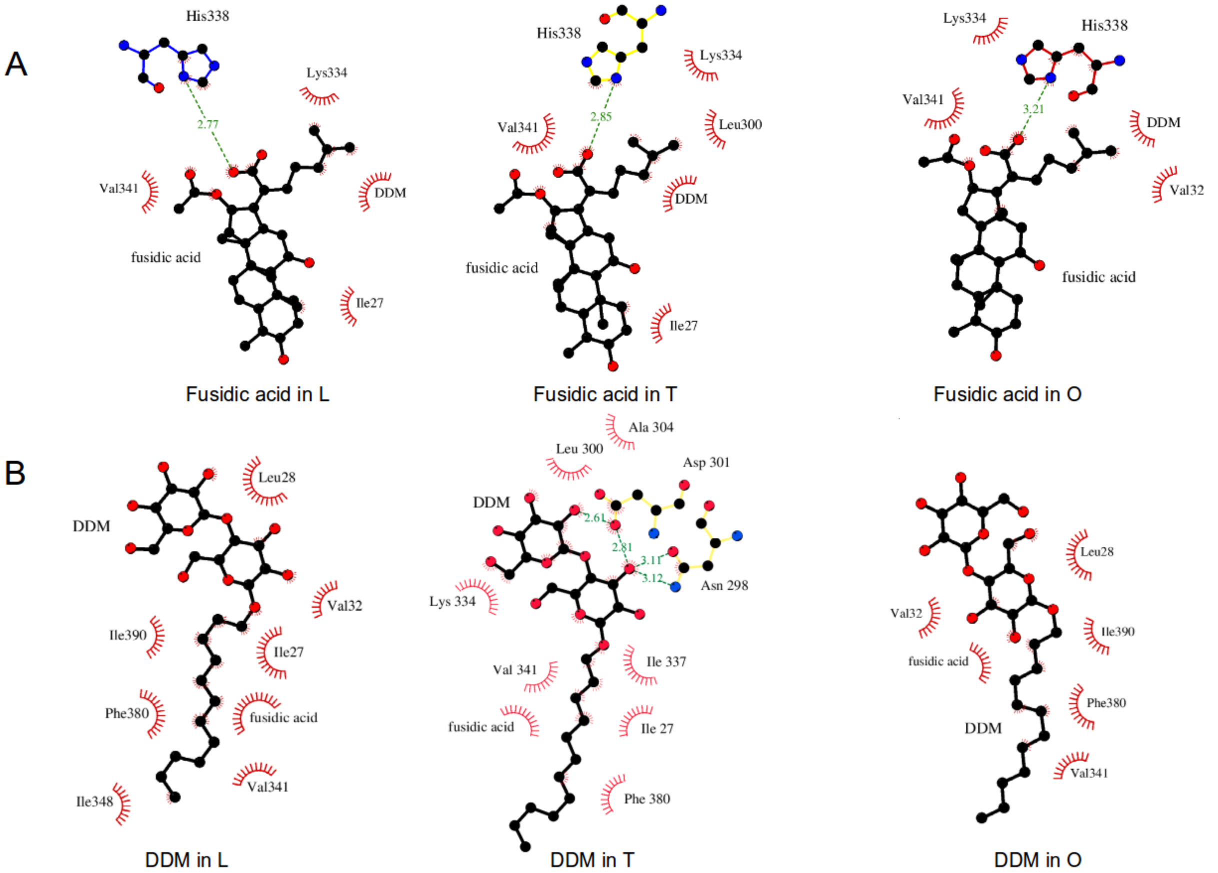

Supplementary Figure 4 - Ligplot representations of fusidic acid and dodecyl-β-D-maltoside binding in the TM1/TM2 area

A Ligand interactions of fusidic acid in the loose (L), tight (T) and open (O) conformer.

B Ligand interaction of dodecyl-β-D-maltoside in the loose (L), tight (T) and open (O) conformer.

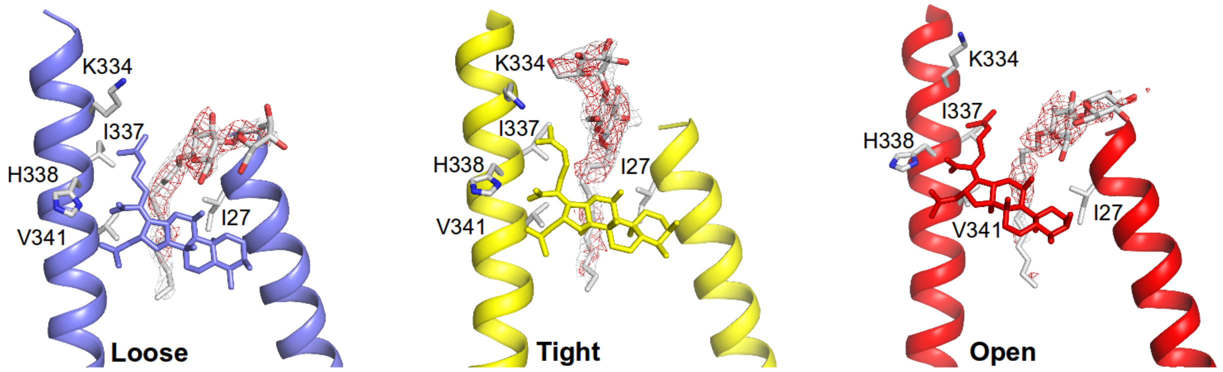

Supplementary Figure 5 - Densities and assignment to dodecyl-β-D-maltoside located at the TM1/TM2 groove

Observed Fo-Fc and 2Fo-Fc SA composite omit maps. Shown are the Fo-Fc densities (at 2.5, 2.5, and 2.0 sigma, in red mesh), 2Fo-Fc densities (at 1.0 sigma, in grey mesh), and DDM assignment at the TM1/TM2 groove of the Loose (colored in blue), Tight (yellow), and Open (red) protomers, respectively.

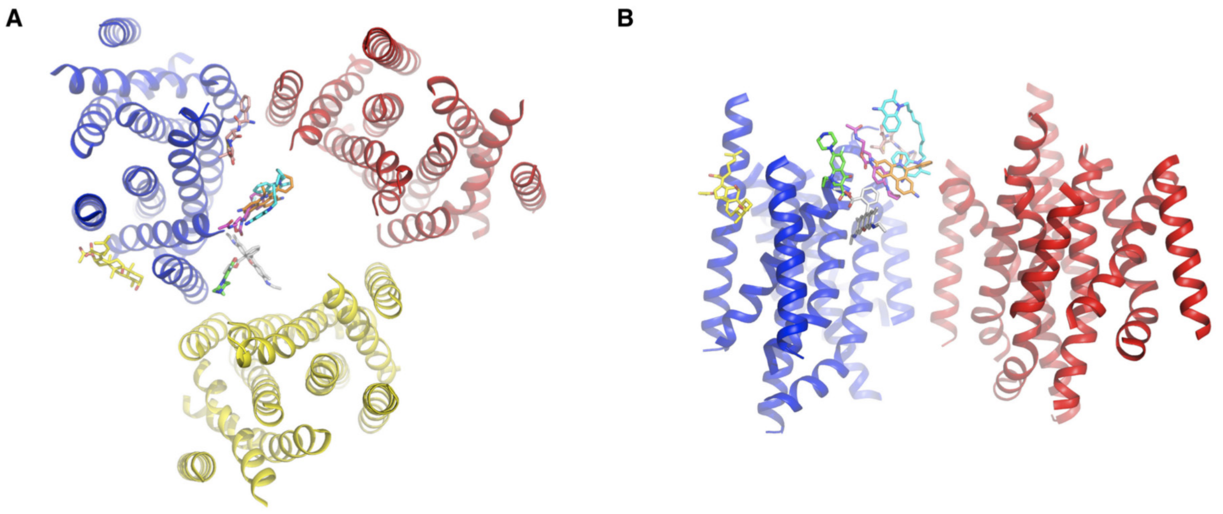

Supplementary Figure 6 - Superimposition of the L protomer of the symmetric AcrB structures with assigned drug molecules bound at the central cavity with the L protomer of the asymmetric fusidic acid AcrB complex structure

Symmetric structures of AcrB comprising one monomer in the asymmetric unit were superimposed on the L monomer (blue) of asymmetric AcrB structure with bound fusidic acid (this work, comprising a trimer in the asymmetric unit, L protomer, blue; T protomer, yellow, O protomer, red). The position of substrates from the symmetric structures are shown in colored stick representation: Rhodamine 6G (white, PDB: 1OY8), Ethidium (orange, PDB: 1OY9), Dequalinium (cyan. PDB: 1OYD), Ciprofloxacin (green, PDB: 1OYE), Linezolid (pink, PDB: 4K7Q), Ampicillin (salmon, PDB: 2RDD). Fusidic acid from this work is shown in yellow stick representation. **A** Top view of the transmembrane domain viewed from the periplasm. **B** View parallel to the membrane plane. Here, the T protomer (yellow in A) has been omitted for clarity.

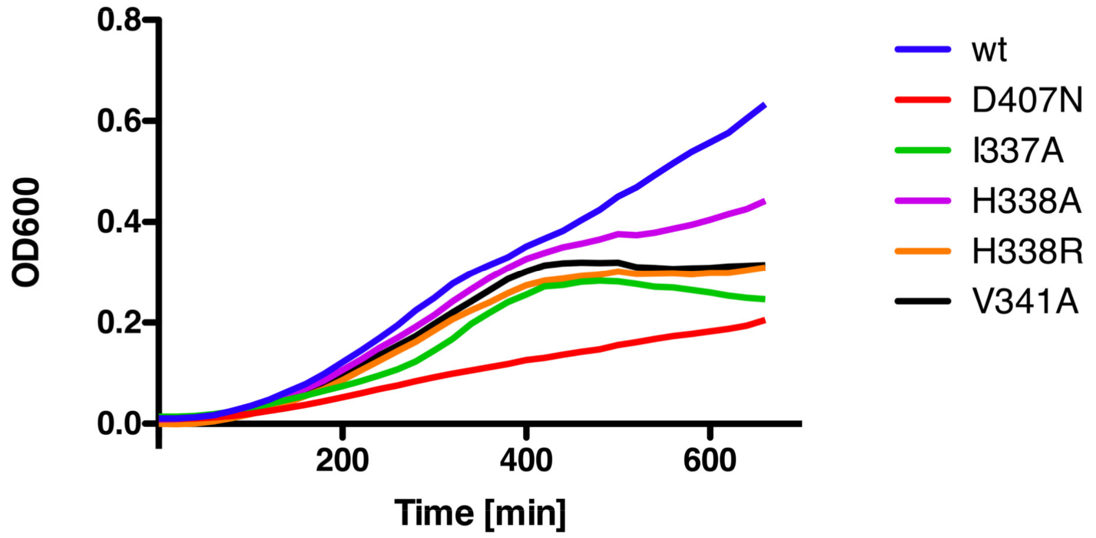

Supplementary Figure 7 - Phenotypic analysis of AcrB wildtype and TM2 mutants in liquid culture containing 60 µg ml^{-1} fusidic acid

Representation of growth curves of *E. coli* BW25113 Δ*acrB* complemented with pET24*acrB* wild type and the indicated mutants on LB supplemented with 60 µg ml^{-1} fusidic acid. The normalized OD_{600} is plotted versus time of growth.

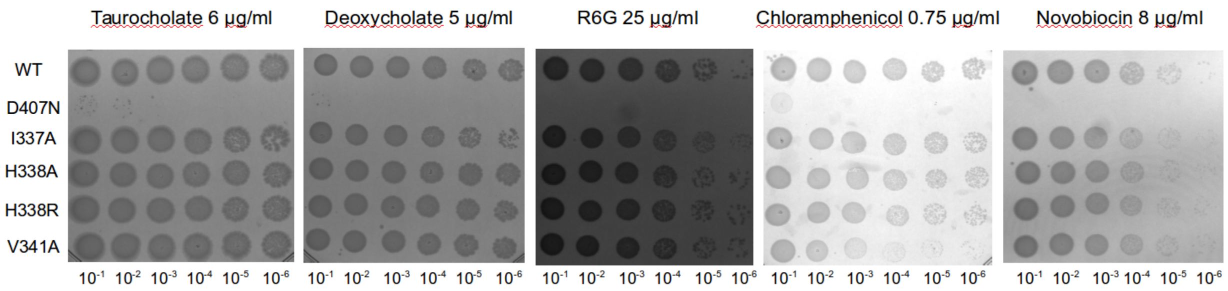

Supplementary Figure 8 - Drug LB-agar plate dilution test with the indicated AcrB variants

E. coli BW25113ΔacrB was complemented with *acrB* wildtype or mutant genes expressed from plasmid. For the assay, colonies of these cells were picked and grown over night in LB_{Kan50} medium at 37 °C, diluted to OD_{600} 10^{-1}–10^{-6}, and 4μl were spotted on an LB_{Kan50} agar plate supplemented with Taurocholate (6 μg/ml), Deoxycholate (5 μg/ml), Rhodamine-6G (25 μg/ml), Chloramphenicol (0.75 μg/ml), or Novobiocin (8 μg/ml). Comparison between wild type and the D407N mutant (deficient in proton translocation) shows in all variants no difference in resistance, with the exception of V341A in the presence of chloramphenicol. Cell dilutions are indicated at the bottom of each plate.

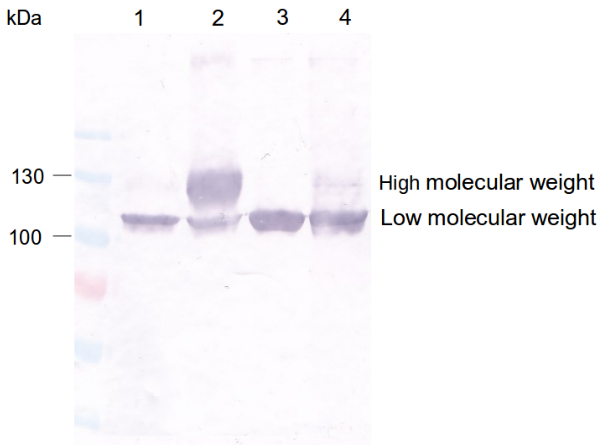

Supplementary Figure 9 - Determination of the extent of MTS-rhodamine cross-linking to AcrB-cl_C338

Membranes containing AcrB-cl_C338 were treated as described in Materials and Methods. Briefly, membranes (80 µl) were washed with cross-linking buffer (CLB), and incubated with either MTS-rhodamine (20 µM) or *N*-ethylmaleimide (NEM, 5 mM) in the absence of AcrB substrate for 15 min on ice. After addition of 420 µl ice-cold CLB excess thiol-reactive reagent was removed by centrifugation. Subsequently, membranes were resuspended in CLB (80 µl) containing 1.5% SDS and methoxypolyethylene glycol maleimide (average molecular weight = ~5000) (MAL-PEG, 3 mM) and incubated at room temperature for 2 h. As a positive control, membranes without prior treatment with NEM or MTS-rhodamine were treated with MAL-PEG in parallel. Membranes without addition of thiol-reactive reagents were used as a negative control. Samples were analyzed by SDS-PAGE and Western blot using α-His antibody as probe. Lane 1: Detection of AcrB-cl_C338 without NEM, MTS-rhodamine, or MAL-PEG treatment; 2: Detection of MAL-PEG only treated AcrB-cl_C338 sample; 3: NEM+MAL-PEG treated sample; 4: MTS-rhodamine + MAL-PEG treated sample.

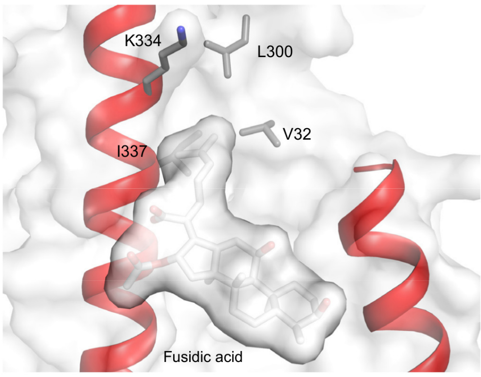

Supplementary Figure 10 - Fusidic acid localization proximal to a hydrophobic area in the AcrB O monomer

In the O conformer, fusidic acid is localized proximal to a hydrophobic pocket, comprising V32, L300, K334 and I337 (as indicated).

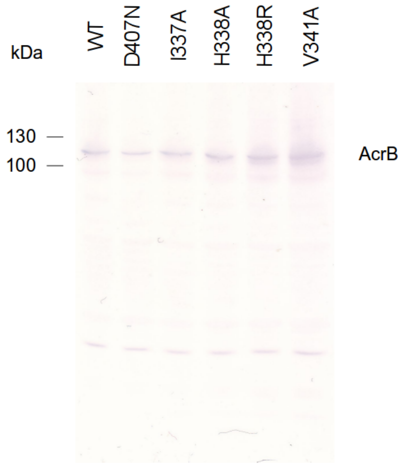

Supplementary Figure 11 – Uncropped image of Figure 4a

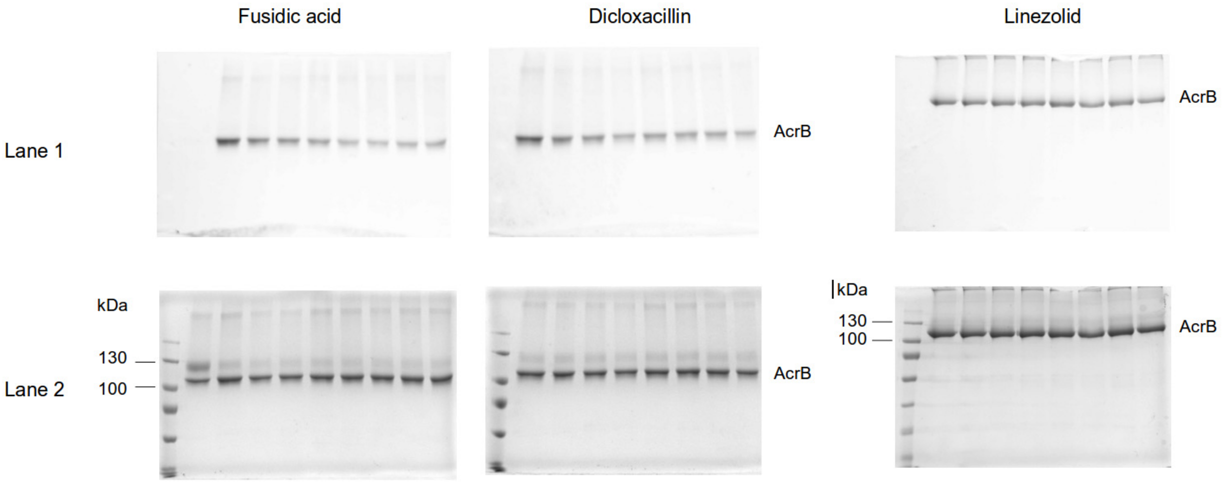

Supplementary Figure 12 – Uncropped images of Figure 5 for in-gel fluorescence (Lane 1) and Coomassie-stained SDS-PAGE analysis of MTS-rhodamine modified AcrB-cl_C338 (Lane 2)

In Lane 2, Row 2 of fusidic acid is AcrB-cl_C338 sample labeled with MAL-PEG. This is a control to show that C338 is able to react with thiol-reactive compounds.

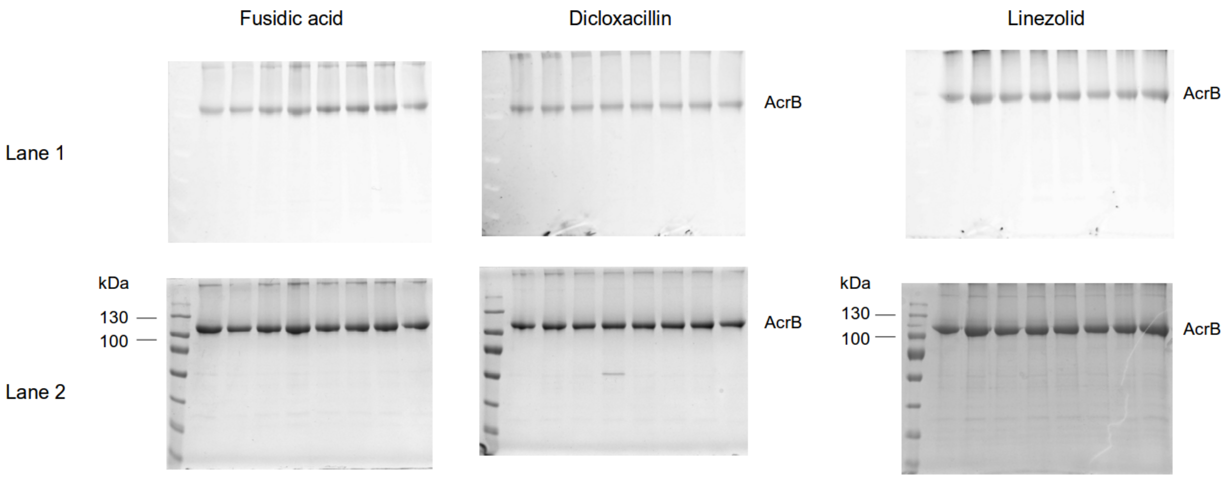

Supplementary Figure 13 – Uncropped images of Figure 5 for in-gel fluorescence (Lane 1) and Coomassie-stained SDS-PAGE analysis of MTS-rhodamine modified AcrB-cl_C14 (Lane 2)

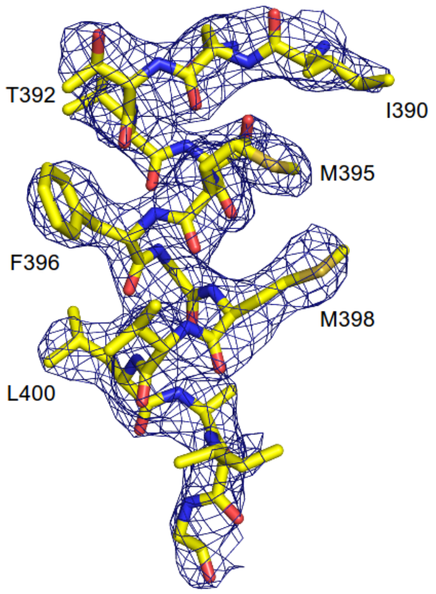

Supplementary Figure 14 – Stereo image of a representative portion of the electron density map of AcrB/DARPins in complex with fusidic acid. Residues 390-403 are shown in stick representation with the 2Fo-Fc electron density map of that region contoured at 1.2σ (blue mesh).

Supplementary Table 1 - Data collection and refinement statistics

	AcrB/DARPins in complex with fusidic acid	AcrB/DARPins in complex with 24-bromofusidic acid
**Data collection**		
Space group	$P2_12_12_1$	$P2_12_12_1$
Cell dimensions		
a, *b*, *c* (Å)	145.65, 163.25, 246.04	145.49, 163.54, 245.10
α; β; γ (°)	90.0, 90.0, 90.0	90.0, 90.0, 90.0
Resolution (Å)	49.12– 2.50 (2.54–2.50)*	49.02– 2.60 (2.64-2.60)
R_{merge}	0.158 (2.290)	0.259 (2.228)
$I / \sigma I$	9.9 (0.8)	10.6 (1.5)
Completeness (%)	100.0 (99.9)	100.0 (100.0)
Redundancy	6.7 (6.7)	13.5 (13.6)
**Refinement**		
Resolution (Å)	2.5	
No. reflections	192130	
R_{work} / R_{free}	22.49 / 26.15	
No. atoms		
Protein	25948	
Ligand/ion	874	
Water	815	
B-factors		
Protein	62.62	
Ligand/ion	108.47	
Water	45.65	
R.m.s. deviations		
Bond length (Å)	0.0069	
Bond angles (º)	1.1048	

Number of crystals for each structure =1. * Highest resolution shell is shown in parenthesis.

Supplementary Table 2 - Primer sequences (PHO: phosphorylated at 5’ end)

Plasmid	Primer sequences
pET24acrB$_{His}$	AcrBfor (5’-GGATCCCATATGCCTAATTTCTTTATCGATC-3’)<br>acrBrev (5’-AAGCTTCTCGAGATGATGATCGACAGTATGGCTG-3’)
pET24acrB-I337A$_{His}$	For_AcrB_I337A (5’-AAGCTTCTCGAGATGATGATCGACAGTATGGCTG-3’)<br>rev_AcrB337-338 (5'PHO-GAACGGCGTGGTGTCATATGGGTAAAC-3)
pET24acrB-H338A$_{His}$	For_AcrB_H338A (5'-GTGAAAATCTCTATTGCCGAAGTGGTTAAAACGC-3')<br>rev_AcrB337-338 (5'PHO-GAACGGCGTGGTGTCATATGGGTAAAC-3)
pET24acrB-H338R$_{His}$	For_AcrB_H338R (5'-GTGAAAATCTCTATTAGAGAAGTGGTTAAAACGC-3')<br>rev_AcrB337-338 (5'PHO-GAACGGCGTGGTGTCATATGGGTAAAC-3)
pET24acrB-V341A$_{His}$	For_AcrB_V341A (5'-CGAAGTGGCAAAAACGCTGGTCGAAGCGATCATCCTCGTGTTCCTGG-3')<br>rev_AcrB341 (5'PHO-TGAATAGAGATCTTCACGAACGGCGTGGTGTCGTATGGG-3')

Supplementary Methods

Abbreviations:

ACN:	Acetonitrile
Br_2:	Bromine liquid
CCl_4:	Carbon tetrachloride
DBU:	1,8-Diazabicyclo[5.4.0]undec-7-ene
DCM:	Dichloromethane
DMF:	N,N-Dimethylformamide
EA	Ethyl acetate
eq.:	Equivalent
EtOH:	Ethanol
HPLC:	High performance liquid chromatography
KH_2PO_4:	Potassium dihydrogen phosphate
LC-MS:	Liquid chromatography mass Spectroscopy
MeOH	Methanol
NaOH:	Sodium hydroxide
Na_2SO_4:	Sodium sulfate
NMR:	Nuclear magnetic resonance
PE	Petroleum ether
R_f:	Retention factor in chromatography
Rt:	Retention time
R.T.:	Room temperature
SCRC:	Sinopharm Chemical Reagent Co., Ltd.
TEA:	Triethylamine
TFA:	Trifluoroacetic acid
TLC:	Thin layer chromatography
Tol.:	Toluene

1. Deliverable of BC1102

1.1 Original deliverables:

Basilea China shall synthesize **24-Bromo-fusidic acid sodium salt BC1111** and deliver 50-100 mg to Basilea Pharmaceutica International Ltd.:

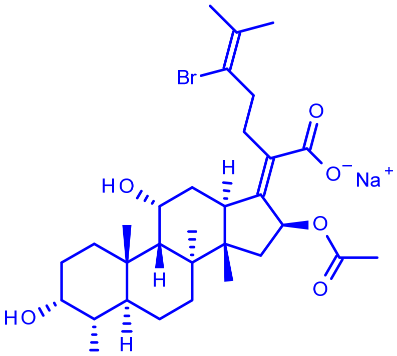

BC1111
BAL0117224

2. Synthesis of BC1111

2.1 General comment

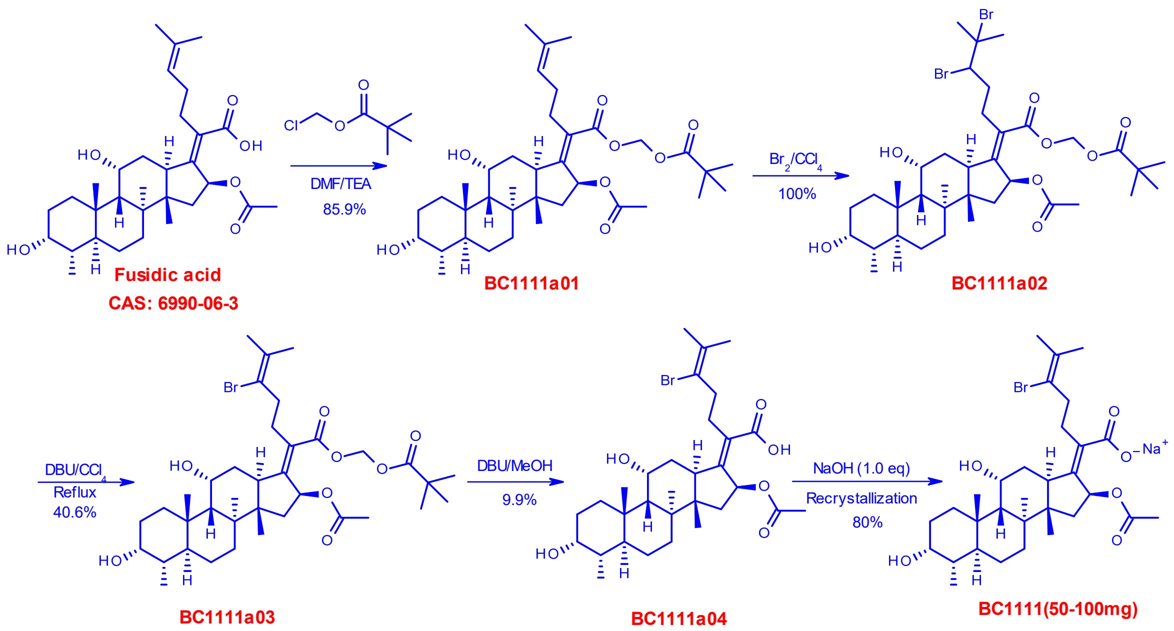

Reference:

Patent: WO2005/007669A1

Instrumentation: All reagents and solvents were of commercial quality and used without further purification unless otherwise noted. The NMR spectra were obtained with a Bruker AVANCE III 400 MHz spectrometer using $CDCl_3$ or DMSO-d_6 as solvent. The chemical shifts are expressed in ppm with $CDCl_3$ or DMSO-d_6 as internal standard. MS (ESI-MS) was recorded on a Waters UPLC H-Class with SQD2 MS spectrometer (Instrument No.03) or Waters 2695/2998 HPLC with SQD MS spectrometer (Instrument No.39). Purity of the products were checked using a Agilent 1200 series instrument (Instrument No.21) equipped with a SunFireTM C18 (3.5μm), 150×4.6 mm column. Peaks were detected at 254 nm or 230 nm or 210 nm absorption. Thin layer chromatography (TLC) was performed using glass plates pre-coated with silica gel GF_{254} with a layer thickness of 0.25 mm purchased from Tsingdao Haiyang Co. Ltd. The conditions for ^{1}H NMR, LC-MS are listed below unless otherwise noted. The HPLC conditions are listed in the analytical data respectively.

NMR analysis

Varian: MercuryPlus-400M

Bruker: AVANCE III 400

Solvent: $CDCl_3$ or DMSO-d_6

Method A:

LC-MS: Instrument No. 03

Instrument: LC/MS system (Instrument No. 33)

Agilent 1100 Series

Components: G1310A/G1315B/G1313A/G1946D

Agilent software (Agilent ChemStations Ver.A. 10.02[1757])

Pump A: Water (prepared using a Millipore ultra pure water system) with 0.1% formic acid

Pump B: ACN (LC-MS grade) with 0.1% formic acid

Column: Agilent SB-C18 (3.5 μm), 4.6 mmx50mm

Column temp: room temperature

Detection: DAD

Flow: 1.5 mL/min

Gradient program:

Step	Time (min)	Phase A (%)	Phase B (%)	Comments
1	0	90	10	Equilibration
2	1	90	10	Linear gradient
3	4	5	95	
4	5.5	5	95	

Method B:

LC-MS:

Instrument No. 39

Instrument: LC/MS system (Instrument No. 39)

Waters 2695/2998 HPLC with SQD MS

Waters Empower2 software

Pump A: Water (prepared using a Millipore ultra pure water system, resistivity: 18.2 MΩ.CM）with 0.1% formic acid

Pump B: ACN (LC-MS grade) with 0.1% formic acid

Column: Atlantis ® T3 (3 μm), 100×4.6 mm

Column temp: 30 °C

Detection: λ= 254 nm, 230 nm and 218 nm/PDA

Flow: 1.0 mL/min

Gradient elution program:

Step	Time (min)	Phase A (%)	Phase B (%)	Comments
1	0	95	5	Equilibration
2	1	95	5	Linear gradient
3	13	5	95	
4	15	5	95	

Method C：

HPLC: Instrument No. 09

HPLC method description:

Instrument: Agilent 1100 Series (HPLC09)
Agilent software (Agilent ChemStations Rev.A. 10.02[1757])

Pump A: Water (prepared using a Millipore ultra pure water system, resistivity: 18.2MΩ.CM) with 10mM K_2HPO_4 (pH=7.2)

Pump B: Acetonitrile (Ourchem®, HPLC grade)

Column: Atlantis® T3 (3μm), 150×4.6 mm

Column temp: R. T.

Sample conc: 0.8mg/mL

Injection vol: 8μL

Detection: λ= 254nm, and 230nm, and 210nm/DAD

Flow: 1.0mL/min

Gradient program:

Step	Time (min)	Phase A (%)	Phase B (%)	Comments

1	0	45	55	Equilibration
2	1	45	55	Linear gradient
3	13	5	95	
4	16.5	5	95	
5	17	95	5	

Method D:

HPLC: Instrument No. 21

HPLC method description:

Instrument: Agilent 1200 Series (HPLC21)
Agilent software (ChemStation for LC 3D system B.04.02 SP1 [208])

Pump A: Water (prepared using a Millipore ultra pure water system, resistivity: 18.2 MΩ.CM) with 0.1% TFA

Pump B: ACN (Ourchem®, HPLC grade) with 0.1% TFA

Column: Prontosil 120-3-C18-SH (3 μm) 150×4.6 mm

Column temp: room temperature

Detection: λ= 230 nm and 210 nm/DAD

Flow: 1.0 mL/min

Gradient program:

Step	Time (min)	Phase A (%)	Phase B (%)	Comments
1	0	98	2	Equilibration
2	5	98	2	Linear gradient
3	18	62	38	
4	25	5	95	
5	30	5	95	

2.2 Synthesis of BAL0117224 (BC1111)

2.2.1 Synthesis of BC1111a01

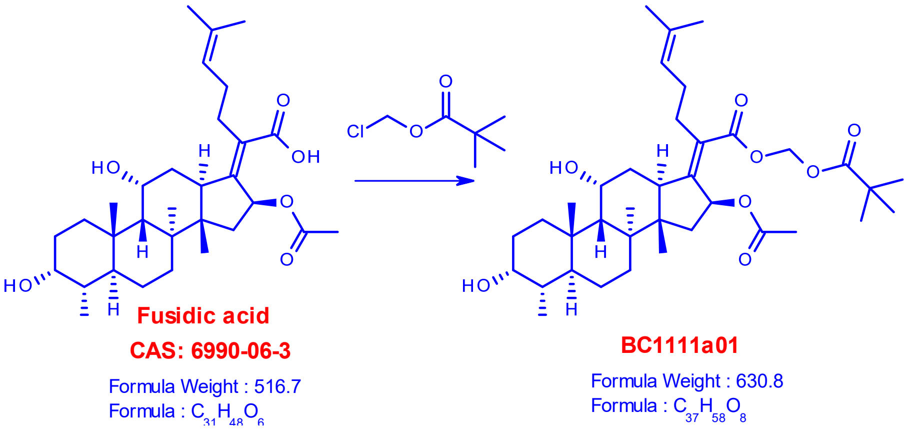

2.2.1.1 General comment

The product was obtained in 85.9% yield after purification.

Reference:

Patent: WO2005/007669A1

2.2.1.2 Experimental Procedure

Lab Journal No.: JIANL-180-1

Experiment No.: JIBC1111a01-001

		Under nitrogen atmosphere, in a 100 mL round-bottomed flask, a mixture of
13.01	g	of Fusidic acid (note 1, 25.18 mmol, 1.0 eq.) and
3.31	g	of TEA (note 2, 32.74 mmol, 1.3 eq.) in
38	mL	of DMF (note 3) was stirred at 25°C for 30 minutes. Then
5.31	g	of Chloromethyl pivalate (note 4, 35.26 mmol, 1.4 eq.) was added dropwise at 25°C over 25 minutes. After addition, the reaction mixture was stirred at 50°C for 18 hours. When TLC showed that the reaction was complete, the mixture was cooled to 25°C and then concentrated under vacuum. The residue was purified by silica gel column chromatography eluting with PE/EA=3/1 (TLC: PE/EA=3/1, R_f=0.25) to give

13.65 g of the product as a white foam.

Product: 13.65 g

Yield: 85.9%

Notes

1. Industrial grade
2. SCRC
3. SCRC
4. SCRC

Analytical Methods

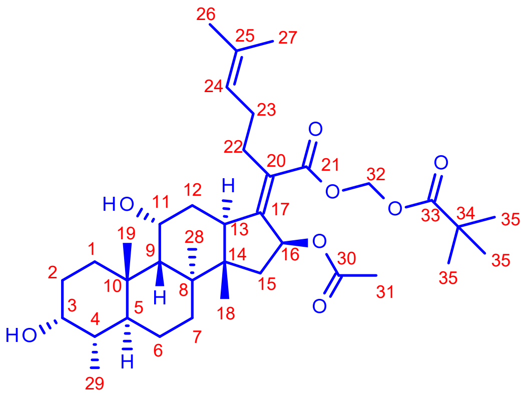

^{1}H NMR (400 MHz, $CDCl_3$) δ ppm: 5.85 (d, *J*=8.4Hz, 1H, H-16), 5.69, 5.78 (AB, *J*=5.6Hz, 2H, H-32), 5.07 (t, *J*=7.2Hz, 1H, H-24), 4.33 (s, 1H, H-3), 3.74 (s, 1H, H-11), 3.04 (d, *J*=11.2Hz, 1H, H-13), 2.35-2.52 (m, 2H, H-2(1H), 12(1H)), 2.25-2.35 (m, 1H, H-4), 2.05-2.20 (m, 4H, H-22, 23), 1.95-2.05 (m, 1H, H-15(1H)), 1.97 (s, 3H, H-31), 1.46-1.90 (m, 8H, H-1(1H), 2(1H), 5, 6, 7(1H), 12(1H), 15(1H)), 1.66 (s, 3H, H-26), 1.58 (s, 3H, H-27), 1.36 (s, 3H, H-28), 1.29 (d, *J*=14.4Hz, 1H, H-9), 1.20 (s, 9H, H-35), 1.00-1.19 (m, 2H, H-1(1H), H-7(1H)), 0.96 (s, 3H, H-18), 0.85-0.95 (m, 6H, H-19, 29)

2.2.1.3 Analytical data

^{1}H NMR analysis: Varian: MercuryPlus-400M

Solvent: $CDCl_3$

NMR of BC1111a01

BC1111 Div.3

Proposed Structure:

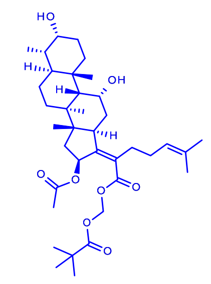

Yes No Uncertain

Solvent:	$CDCl_3$
Producer:	JIAN L
Analyst:	Alain
Date:	20131106
Analysis No:	AN1301013192
Comment:	OA01
ELN No:	JIANL-180-1

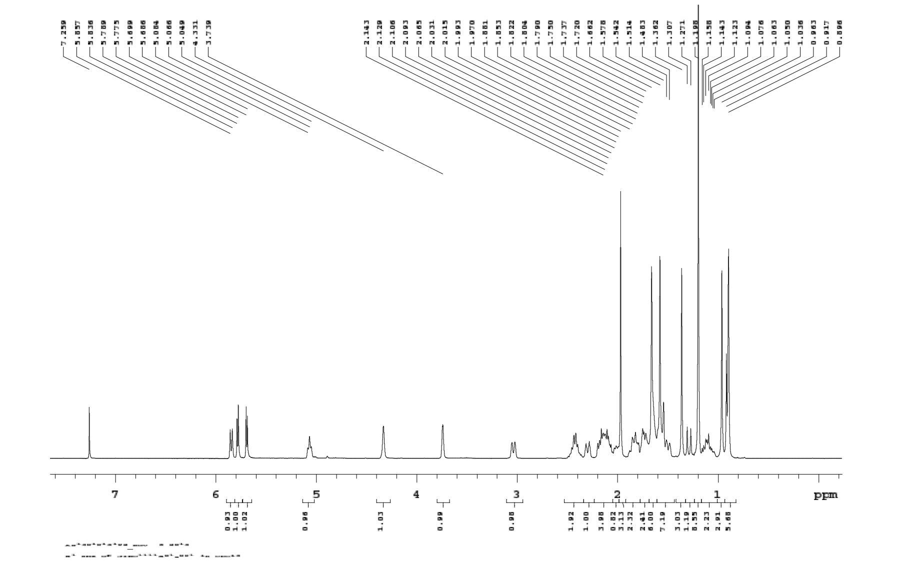

ANALYTICAL DATA SHEET (Basilea China) JI/PD-ANA-002[01]

2.2.2 Synthesis of BC1111a02

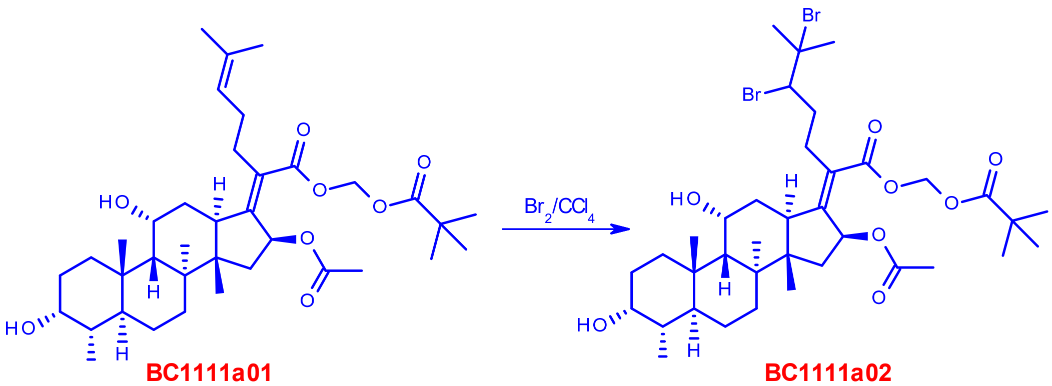

BC1111a01

Formula Weight : 630.8

Formula : $C_{37}H_{58}O_8$

BC1111a02

Formula Weight : 790.7

Formula : $C_{37}H_{58}Br_2O_8$

2.2.2.1 General comment

The experimental result indicated that the product was labile over silica gel. Thus the crude product was used directly in the next step without further purification after work up. The crude product was analyzed by LCMS but it coudn’t be ionized.

Reference:

Patent: WO2005/007669A1

2.2.2.2 Experimental Procedure

Lab Journal No.: JIANL-189-1

Experiment No.: JIBC1111a02-003

		Under nitrogen atmosphere, in a 250 mL round-bottomed flask,
7.15	g	of BC1111a01 (note 1, 11.33 mmol, 1.0 eq.) was dissolved in
40	mL	of CCl_4 (note 2) and the resulting mixture was cooled to -10°C. Then a solution of
1.99	g	of Br_2 (note 3, 12.47 mmol, 1.1 eq.) in
40	mL	of CCl_4 (note 2) was dropwise added in the course of 1 hour with continuous stirring at -10°C. After addition, the resulting reaction mixture was further stirred at -10°C for 1 hour. When TLC showed that the reaction was complete, the mixture was concentrated under vacuum to give the crude product as a light yellow foam. The crude product was used directly in the next step without further purification.

Product: 8.95 g

Yield: 100%

Notes

1. JIANL-180-1
2. SCRC, dried over 4 Å molecular sieve
3. SCRC

2.2.3 Synthesis of BC1111a03

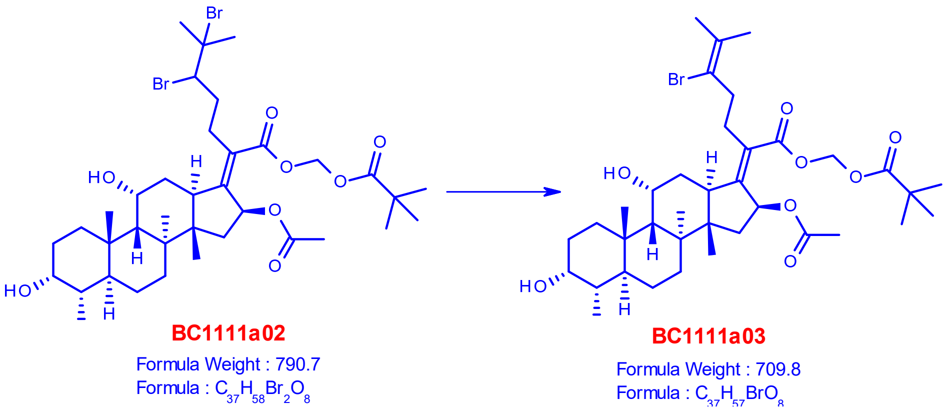

2.2.3.1 General comment

The product was prepared in about 40% yield after purification by silica gel column chromatography.

Reference:

Patent: WO2005/007669A1

2.2.3.2 Experimental Procedure

Lab Journal No.: JIANL-185-1

Experiment No.: JIBC1111a03-002

		Under Argon atmosphere, to a solution of
3.78	g	of BC1111a02 (note 1, 4.78 mmol, 1.0 eq.) in
50	mL	of CCl_4 (note 2) was added
1.46	g	of DBU (note 3, 9.56 mmol, 2.0 eq.). The mixture was refluxed at 77°C for 16 hours. Then the reaction mixture was filtered through Celite. The filter cake was washed with
100	mL	of PE and
100	mL	of EA successively. The combined filtrate and washings were concentrated under reduced pressure. The residue was purified by silica gel column chromatography eluting with PE/EA=5/1 (TLC: PE/EA=3/1, R_f=0.25) to give
1.29	g	of the product as a light yellow foam.

Product: 1.29 g

Yield: 38.1%

Notes

1. JIANL-184-1
2. SCRC, anhydrous
3. SY-001471

Analytical Methods

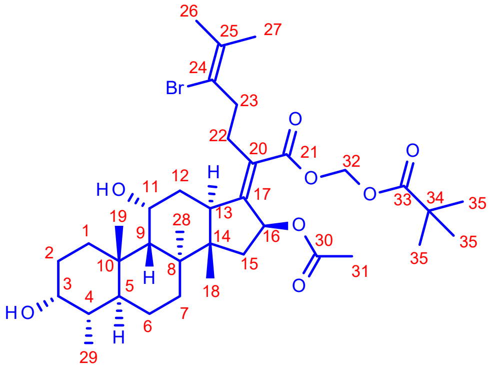

^{1}H NMR (400 MHz, $CDCl_3$) δ ppm: (the sample was not clean) 5.85 (m, 1H, H-16), 5.68, 5.82 (AB, 2H, H-32), 4.35 (s, 1H, H-3), 3.72 (s, 1H, H-11), 3.02-3.12 (m, 1H, H-13), 2.06-2.22, 2.35-2.58 and 2.58-2.75 (3m, 7H, H-2(1H), 4, 12(1H), 22, 23), 1.92-2.00 (m, 4H, H-15(1H), 31), 1.45-1.65 and 1.65-1.92 (m, 8H, H-1(1H), 2(1H), 5, 6, 7(1H), 12(1H), 15(1H)), 1.85 (s, 3H, H-26), 1.75 (s, 3H, H-27), 1.36 (s, 3H, H-28), 1.25-1.32 (m, 1H, H-9), 1.24 (s, 9H, H-35), 1.03-1.20 (m, 2H, H-1(1H), 7(1H)), 0.96 (s, 3H, H-18), 0.85-0.95 (m, 6H, H-19, 29)

2.2.3.3 Analytical data

^{1}H NMR analysis: Varian: MercuryPlus-400M

Solvent: $CDCl_3$

NMR of BC1111a03

basilea PHARMACEUTICA

BC1111 Div.3

Proposed Structure:

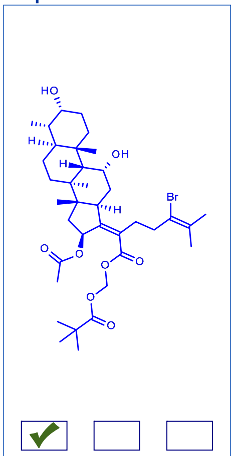

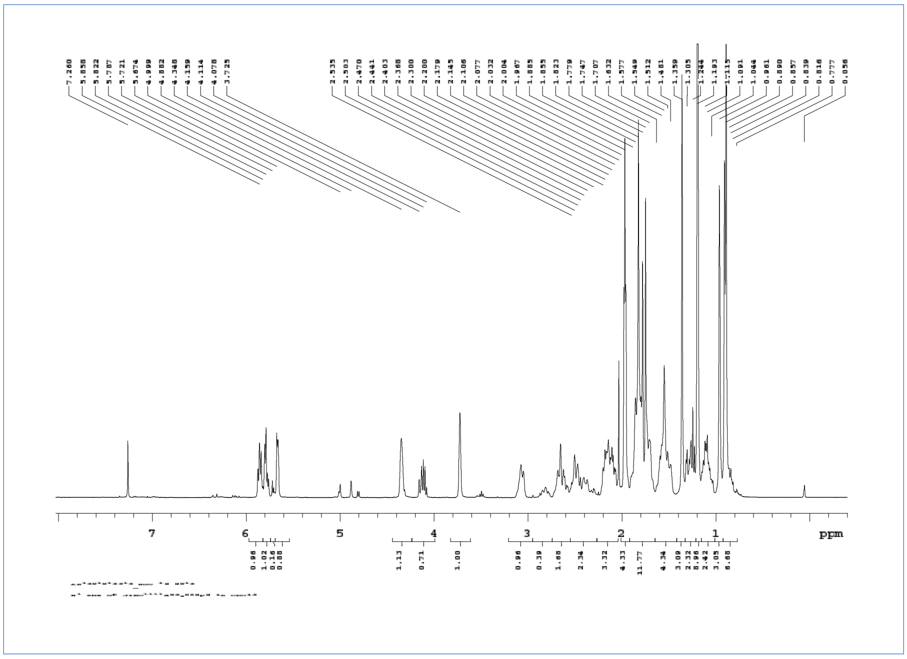

**Solvent:**	$CDCl_3$
**Producer:**	JIAN L
**Analyst:**	Alain
**Date:**	20131112
**Analysis No:**	AN1301013313
**Comment:**	OA01
**ELN No:**	JIANL-185-1

ANALYTICAL DATA SHEET (Basilea China) JI/PD-ANA-002[01]

2.2.4 Synthesis of BC1111a04

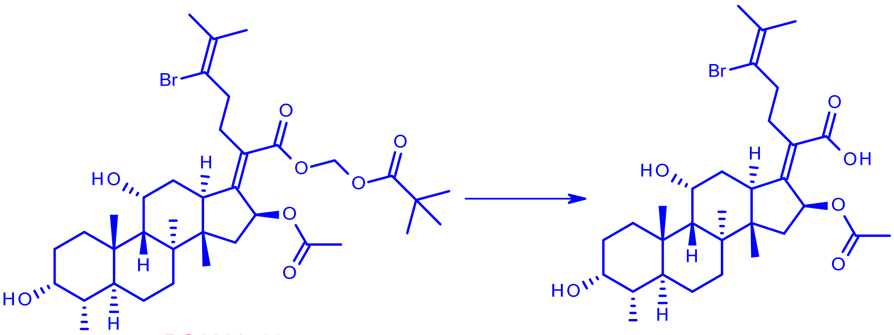

BC1111a03
Formula Weight : 709.8
Formula : $C_{37}H_{57}BrO_8$

BC1111a04
Formula Weight : 595.6
Formula : $C_{31}H_{47}BrO_6$

2.2.4.1 General comment

A main impurity with similar polarity to the product couldn't be removed by silica gel column chromatography. Then the product was tried to be purified by recrystallization using EA/Toluene as solvent according to the patent but failed. Later, the product was treated with DCM/MeOH/PE and most of the impurity was precipitated and removed by filtration.

After then the filtrate was concentrated to give the product with better purity which was further purified by preparative HPLC.

Reference:

Patent: WO2005/007669A1

2.2.4.2 Experimental Procedure

Lab Journal No.: JIANL-191-001

Experiment No.: JIBC1111a04-003

		Under Argon atmosphere, to a solution of
3.26	g	of BC111a03 (note 1, 4.59 mmol, 1.0 eq.) and
0.32	g	of DBU (note 2, 2.11 mmol, 0.46 eq.) in
40	mL	of MeOH (note 3) was added dropwise a mixed solution of
25	mL	of MeOH (note 3) and
25	mL	of H_2O over 1 hour. Then the mixture was stirred at 20°C for 5 hours. Then the mixture was acidified with 1.0 M aqueous solution of KH_2PO_4 until pH=4-5. The resulting mixture was extracted with
100	mLX3	of EA. The combined organic phases were washed with
60	mL	of H_2O and
60	mLX2	of brine successively, dried over Na_2SO_4, and concentrated under vacuum. The residue was purified by silica gel column chromatography (Eluent: PE/EA/HCOOH=50/50/0.5) to give
2.30	g	of the product as white solid. The resulting solid was dissolved in
5	mL	of DCM and
1	mL	of MeOH followed by dropwise addition of
30	mL	of PE at 20°C. Most of the impurity was precipitated and then removed by filtration. Then the filtrate was concentrated to give
0.70	g	of the desired product with better purity which was further purified by preparative HPLC (note 4) to give
0.27	g	of the pure product as a white solid.

Product: 270 mg

Yield: 9.9%

Notes

1. JIANL-190-1
2. SY-001471
3. SCRC
4. Prep. HPLC condition:

   Pump A: Water with 0.1% TFA

   Pump B: Acetonitrile

   Column: Atlantis® Prep T3 OBD™ 19×150mm 5μm

   Column temp: rt

   Detection: λ= 210nm

   Flow: 14 mL/min

   Gradient program:

Step	Time (min)	Phase A (%)	Phase B (%)	Comments
1	0.0	80	20	Linear gradient
2	11.0	40	60	
3	11.1	40	60	
4	15.0	0	100	

Analytical Methods

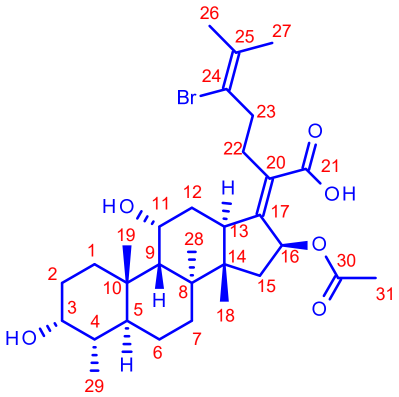

^{1}**H NMR** (400 MHz, $CDCl_3$) δ ppm: (some EA was left in the sample) 8.02 (s, 1H, COOH), 5.88 (d, *J*=8.4Hz, 1H, H-16), 4.38 (s, 1H, H-3), 3.76 (s, 1H, H-11), 3.08 (d,

J=11.2Hz, 1H, H-13), 2.48-2.80 (m, 4H, H-22, 23), 2.00-2.22 (m, 4H, H-4, 2(1H), 12(1H), 15(1H)), 1.97 (s, 3H, H-31), 1.83 (s, 3H, H-26), 1.76 (s, 3H, H-27), 1.78-1.93, 1.70-1.78 and 1.46-1.65 (3m, 8H, H-1(1H), 2(1H), 5, 6, 7(1H), 12(1H), 15(1H)), 1.37 (s, 3H, H-28), 1.26-1.35 (m, 1H, H-9), 1.03-1.18 (m, 2H, H-1(1H), 7(1H)), 0.98 (s, 3H, H-18), 0.87-0.95 (m, 6H, H-19, 29)

^{13}C NMR (400 MHz, $CDCl_3$) δ ppm: 173.6 (C-21), 170.3 (C-30), 151.8 (C-17), 130.9 (C-25), 127.7 (C-20), 119.7 (C-24), 74.0 (C-16), 71.1 (C-3), 67.7 (C-11), 48.9 (C-9), 48.2 (C-14), 44.0 (C-15), 39.0, 38.4 and 37.4 (C-5, 8 13), 36.3, 36.1 and 35.2 (C-4, 12, 23), 31.3, 29.5 and 29.2 (C-1, 2, 7), 27.4 (C-10), 24.8, 23.2 and 22.8 (C-6, 19, 22), 20.6, 20.5 and 20.2 (C-18, 28, 31), 17.3 (C-26, 27), 15.5 (C-29)

2.2.4.3 Analytical data

^{1}H NMR analysis: Varian: MercuryPlus-400M

Solvent: $CDCl_3$

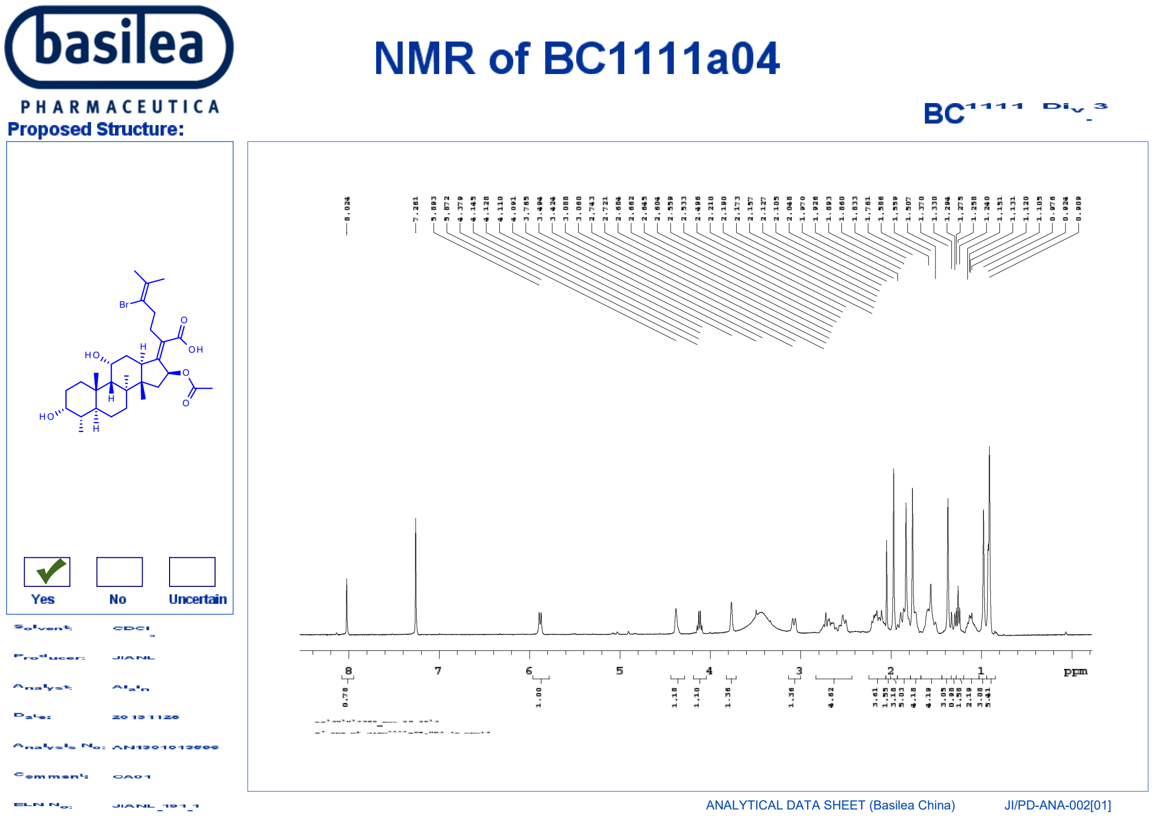

^{13}C NMR analysis: Bruker: AVANCE III 400

Solvent: $CDCl_3$

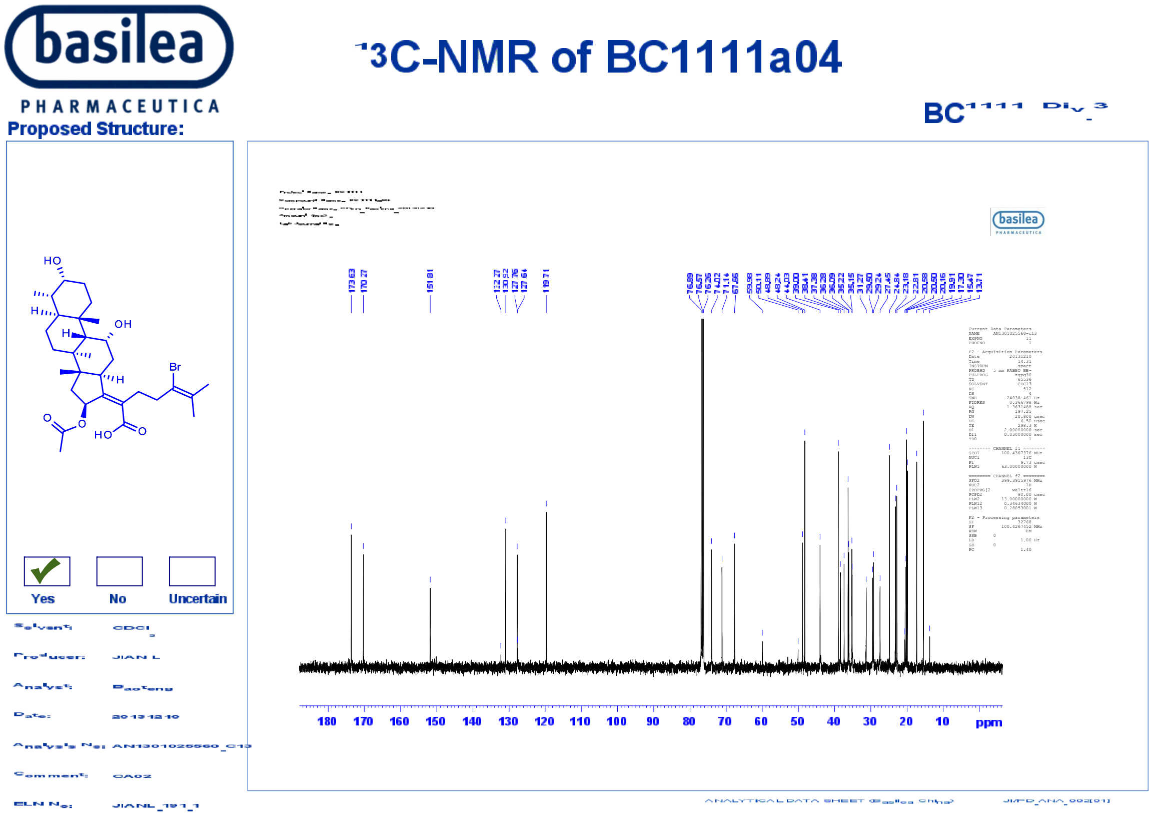

2.2.5 Synthesis of BAL0117224 (BC1111)

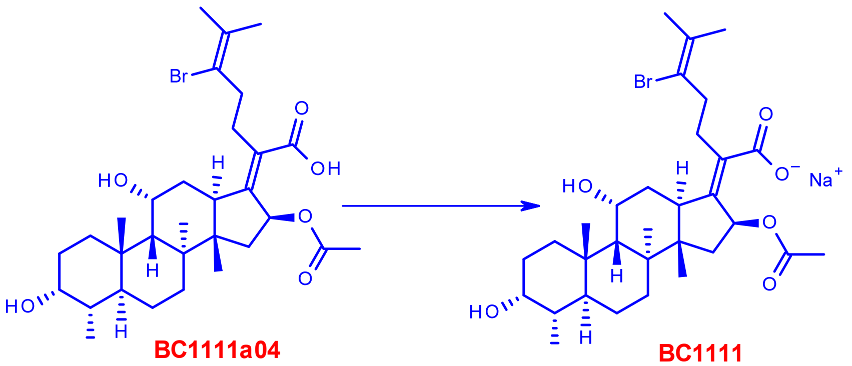

BC1111a04
Formula Weight : 595.6
Formula : $C_{31}H_{47}BrO_6$

BC1111
Formula Weight : 617.6
Formula : $C_{31}H_{46}BrNaO_6$

2.2.5.1 General comment

Reference:

Patent: WO2005/007669A1

2.2.5.2 Experimental Procedure

Lab Journal No.: JIANL-199-1

Experiment No.: JIBC1111-001

		To a solution of
80	mg	of BC1111a04 (note 1, 0.13 mmol, 1.0 eq.) in
1.0	mL	of MeOH (note 2) was added a solution of
0.13	mL	of 1.0 M aqueous solution of NaOH (note 3, 0.13 mmol, 1.0 eq.) at 20°C. The resulting solution was concentrated and the residue was dissolved in
5	mL	of EtOH (note 4) followed by dropwise addition of
5	mL	of EA (note 5) at 20°C, but no precipitate formed. Then the resulting mixture was concentrated under reduced pressure until about 2 mL of oily residue was left. Then
5	mL	of EA (note 5) was added at 20°C and a lot of white precipitate formed. The white solid was collected by filtration and then dissolved in
1	mL	of ACN and
5	mL	of H_2O. The resultant mixture was lyophilized to afford
67	mg	of the product as a white solid.

Product: 67 mg

Yield: 80.7%

Purity: 92.3% (210 nm)

Notes

1. JIANL-191-1
2. SCRC
3. SCRC
4. SCRC
5. SCRC

Analytical Methods

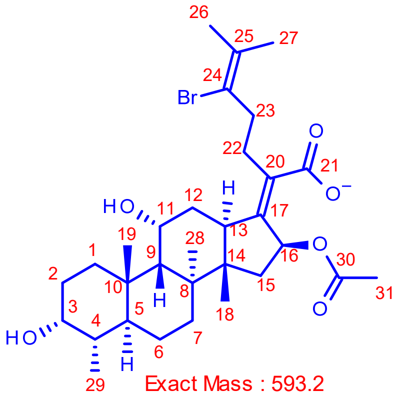

^{1}H NMR (400 MHz, DMSO-d_6+D_2O) δ ppm: 5.61 (d, *J*=8.0Hz, 1H, H-16), 4.11 (s, 1H, H-3), 3.50 (s, 1H, H-11), 2.80 (d, *J*=11.2Hz, 1H, H-13), 2.65-2.75 (m, 1H, H-4), 2.40-2.50, 2.13-2.38 and 2.00-2.10 (3m, 6H, H-22, 23, 2(1H), 12(1H)), 1.85 (s, 3H, H-31), 1.75 and 1.77 (2s, 6H, H-26, 27), 1.82-1.92 and 1.56-1.70 (2m, 4H, H-15, 2(1H), 12(1H)), 1.28-1.55 and 0.93-1.10 (2m, 8H, H-1, 5, 6, 7, 9), 1.24 (s, 3H, H-28), 0.87 (s, 3H, H-18), 0.80 (s, 3H, H-19), 0.78 (d, *J*=6.4Hz, 3H, H-29)

MS m/z (-ESI): 593.4/595.6 ($[M-H]^-$, 68%/100%).

HPLC: Rt =22.86 min (Purity: 88.5% at 254 nm, 91.5% at 230 nm, and 92.3% at 210 nm).

2.2.5.3 Analytical data

^{1}H NMR analysis: Bruker: AVANCE III 400

Solvent: d_6-DMSO

NMR of BAL0117224-001-001 (BC1111)

BC1111 Div_3

Proposed Structure:

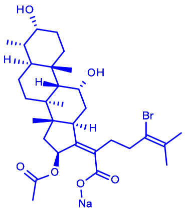

☑ Yes ☐ No ☐ Uncertain

Solvent: DMSO_d6
Producer: JIAN L
Analyst: Baoteng
Date: 20131204
Analysis No: AN1301025428
Comment: QA02
ELN No: JIANL_199_1

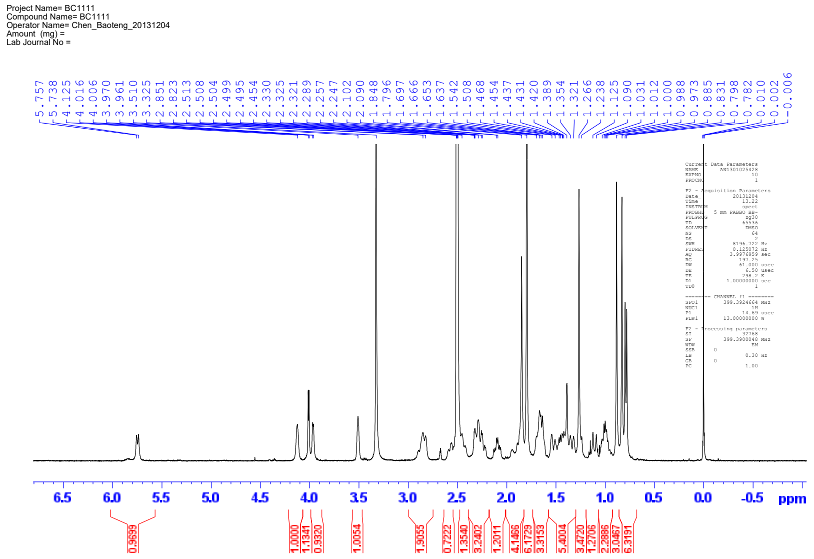

NMR of BAL0117224-001-001 (BC1111)

BC1111 Div_3

Proposed Structure:

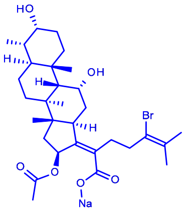

☑ Yes ☐ No ☐ Uncertain

Solvent: DMSO_d6+D2O
Producer: JIAN L
Analyst: Baoteng
Date: 20131204
Analysis No: AN1301025429
Comment: QA02
ELN No: JIANL_199_1

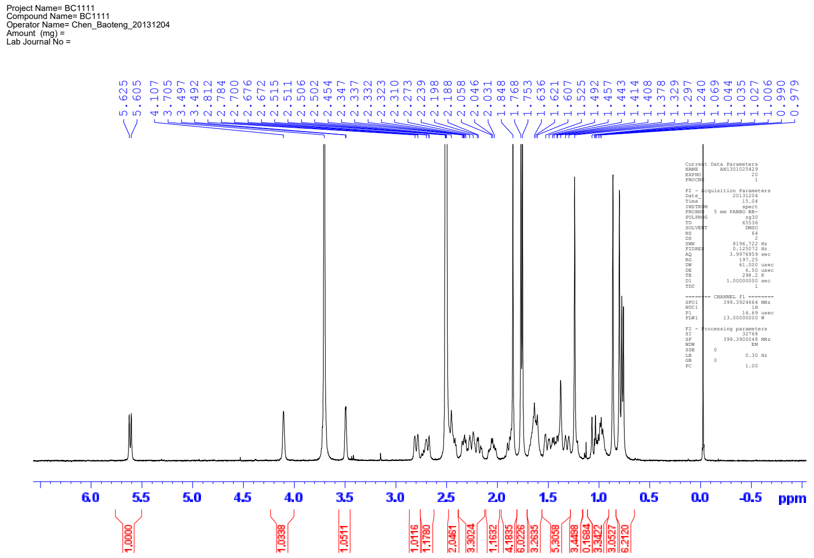

LC-MS analysis: Method A

LCMS of BAL0117224-001-001 (BC1111)

BC1111_Div_3

Proposed Structure:

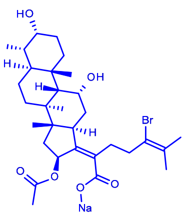

☑ Yes ☐ No ☐ Uncertain

Exact Mass: 616.2

Producer: JIAN L

Analyst: Wilbur Zhao

Date: 20131204

Analysis No: AN1303030276

Comment: OA02

ELN No: JIANL_199_1

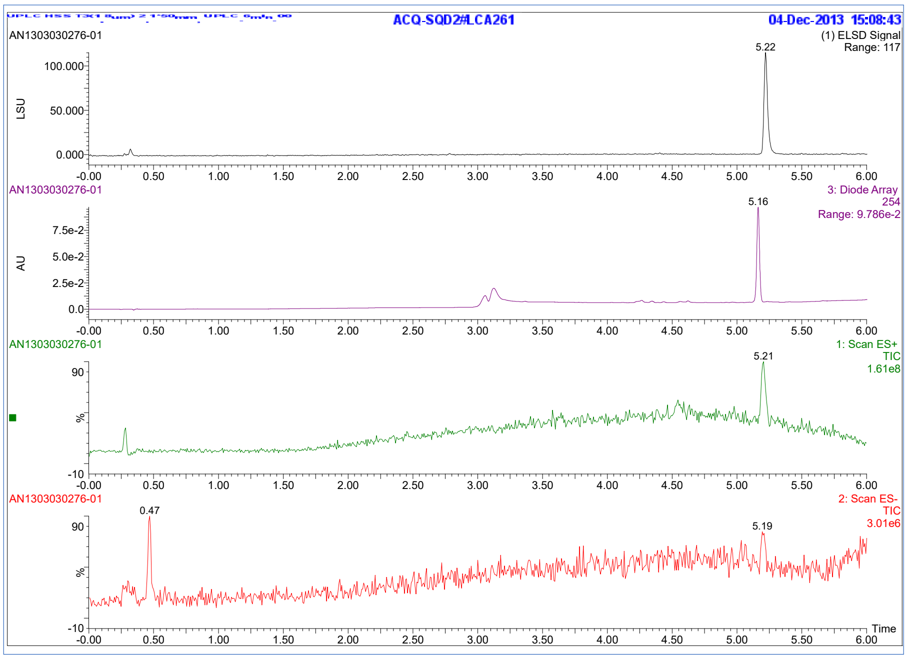

basilea PHARMACEUTICA

LCMS of BAL0117224-001-001 (BC1111)

BC1111 Div 3

Proposed Structure:

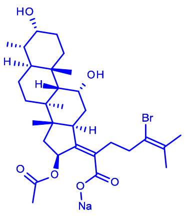

☑ Yes ☐ No ☐ Uncertain

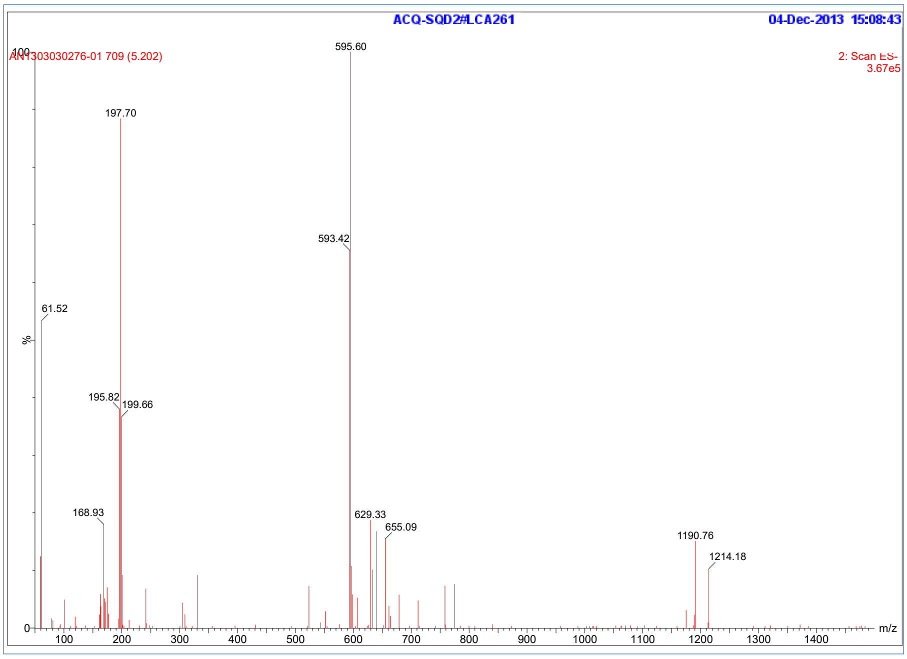

Exact Mass: 616.2

Producer: JIAN L

Analyst: Wilbur Zhao

Date: 20131204

Analysis No: AN1303030276

Comment: QA02

ELN No: JIANL_199_1

HPLC: Method D

HPLC of BAL0117224-001-001 (BC1111)

PHARMACEUTICA

BC1111 Div_3

Proposed Structure:

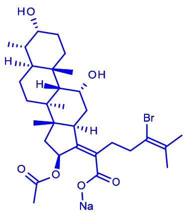


```
Data file  :    C:\CHEM32\1\DATA\20131204\AN1302212940.D
Injection Date: 12/4/2013     3:42:48 PM

Sample Name:    BC1111
Concentration:  0.400mg/ml
Inj. Vol.  :          8 µl 03:42:->
Acq. Method :   C:\CHEM32\1\METHODS\89-4TFA-2.M
Column :          sun fire 4.6*150 3.5
```

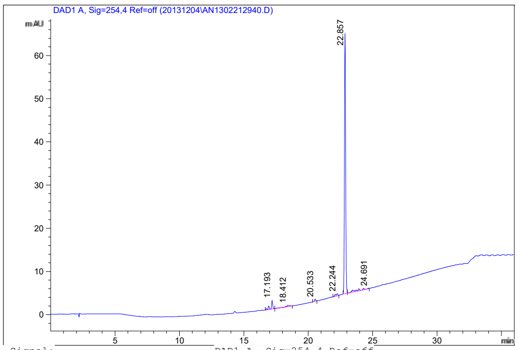

Signal: DAD1 A, Sig=254,4 Ref=off

Area Percent Report

R.T. [min]	Width [min]	Area	Area %
17.193	0.156	19.040	4.397
18.412	0.268	5.184	1.197
20.533	0.100	3.003	0.693
22.244	0.208	6.521	1.506
22.857	0.106	383.096	88.474
24.691	2.150	16.161	3.732
:		433.006	100.000

HPLC-21 Thu, 5. Dec. 2013 11:04:43 am Page 1 of 1

Solvent: ACN+H2O

Producer: JIAN L

Analyst: Caroline

Date: 20131204

Analysis No: AN1302212940

Comment: OA02

ELN No: JIANL_199_1

HPLC of BAL0117224-001-001 (BC1111)

PHARMACEUTICA

BC1111 Div_3

Proposed Structure:

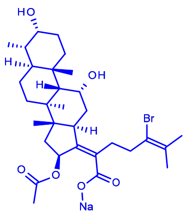


```
Data file  :    C:\CHEM32\1\DATA\20131204\AN1302212940.D
Injection Date: 12/4/2013     3:42:48 PM

Sample Name:    BC1111
Concentration:  0.400mg/ml
Inj. Vol.  :          8 µl 03:42:->
Acq. Method :   C:\CHEM32\1\METHODS\89-4TFA-2.M
Column :          sun fire 4.6*150 3.5
```

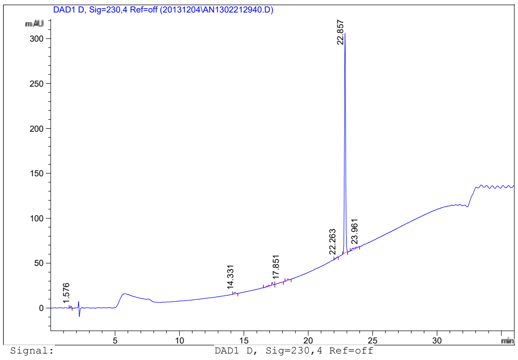

Signal: DAD1 D, Sig=230,4 Ref=off

Area Percent Report

R.T. [min]	Width [min]	Area	Area %
1.576	0.102	15.610	0.924
14.331	0.168	17.997	1.065
17.194	0.188	39.711	2.351
17.851	0.158	4.403	0.261
18.415	0.119	11.127	0.659
22.263	0.223	10.961	0.649
22.857	0.105	1546.216	91.524
23.961	2.119	43.383	2.568
:		1689.407	100.000

HPLC-21 Thu, 5. Dec. 2013 11:05:33 am Page 1 of 1

Solvent: ACN+H2O

Producer: JIAN L

Analyst: Caroline

Date: 20131204

Analysis No: AN1302212940

Comment: OA02

ELN No: JIANL_199_1

HPLC of BAL0117224-001-001 (BC1111)

BC1111 Div_3

Proposed Structure:

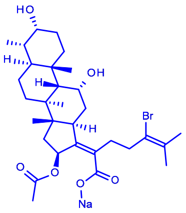

Solvent: ACN+H2O

Producer: JIAN L

Analyst: Caroline

Date: 20131204

Analysis No: AN1302212940

Comment: OA02

ELN No: JIANL_199_1

```
Data file  :     C:\CHEM32\1\DATA\20131204\AN1302212940.D
Injection Date:  12/4/2013     3:42:48 PM

Sample Name:     BC1111
Concentration:   0.400mg/ml
Inj. Vol.  :           8 µl 03:42:->
Acq. Method :    C:\CHEM32\1\METHODS\89-4TFA-2.M
Column :           sun fire 4.6*150 3.5
```

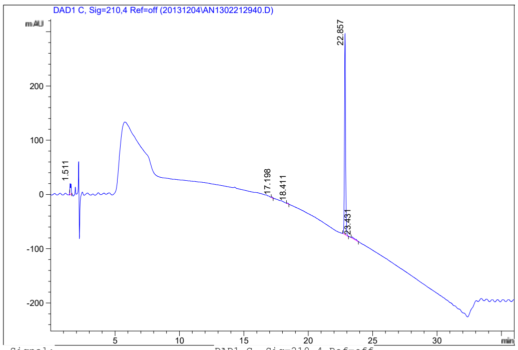

Signal: DAD1 C, Sig=210,4 Ref=off

Area Percent Report

R.T. [min]	Width [min]	Area	Area %
1.511	0.100	124.114	4.846
17.198	0.103	13.130	0.513
18.411	0.105	10.371	0.405
22.857	0.106	2363.891	92.289
23.431	0.316	49.899	1.948
:		2561.405	100.000

HPLC-21 Thu, 5. Dec. 2013 11:06:35 am Page 1 of 1

3. Conclusion

The delivered target compounds were listed in the following table.

Compounds	Batch No.	Analytical No.
BAL0117224-001-001<br>(BC1111)	JIANL-199-1	^{1}HNMR: AN1301025428<br>^{1}HNMR: AN1301025429<br>HPLC: AN1302212940<br>LCMS: AN1303030276
BAL0117224-001-002<br>(BC1111)	JIANL-206-1	^{1}HNMR: AN1301025575<br>^{1}HNMR: AN1301025576<br>HPLC: AN1302213052<br>LCMS: AN1303030304

4. Batch tracking

4.1 Synthesis of BC1111a01

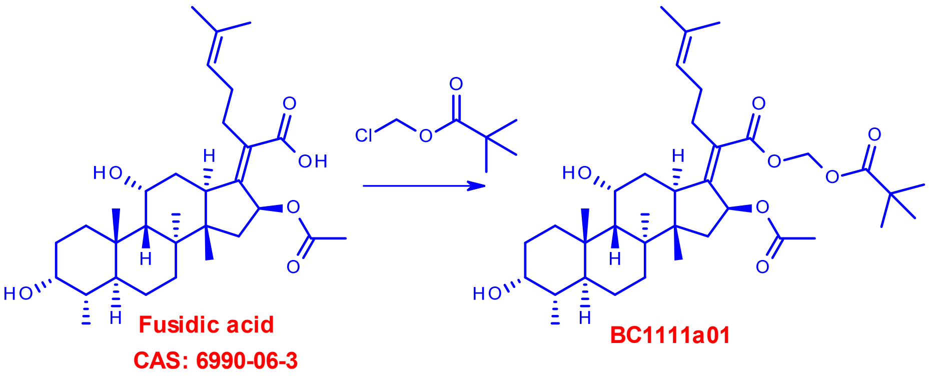

Reaction conditions:

Experiment No.	Date	Fusidic acid			Chloromethyl pivalate		TEA		DMF	Temp.	Time
		Batch No.	g	mmol	g	mmol/eq.	g	mmol/eq.	mL	ºC	hr
JIBC1111a01-001	11/3/2013	industrial grage	13.01	25.18	5.31	35.26/1.4	3.31	32.74/1.3	38	50	18

Results:

Experiment No.	Batch No.	Date	crude product					pure product				
			Analytical date			Qty.	Yield	Analytical date			Qty.	Yield
			HPLC	LCMS	NMR	mg	%	HPLC	LCMS	NMR	g	%
JIBC1111a01-001	JIANL-180-1	11/4/2013								AN13010 13192	13.65	85.9

4.2 Synthesis of BC1111a02

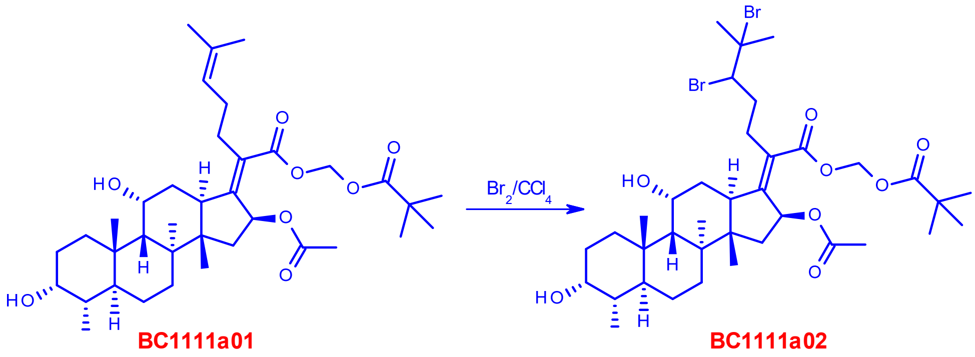

Reaction conditions:

Experiment No.	Date	BC1111a01			Br_2		CCl_4	Temp.	Time
		Batch No.	g	mmol	g	mmol/eq.	mL	ºC	hr
JIBC1111a02-001	11/5/2013	JIANL-180-1	3.01	4.77	0.84	5.25/1.1	40	0	2
JIBC1111a02-002	11/11/2013	JIANL-180-1	3.02	4.78	0.84	5.26/1.1	40	-10	2
JIBC1111a02-003	11/21/2013	JIANL-180-1	7.15	11.30	2.00	12.5/1.1	80	-10	2

Results:

Experiment No.	Batch No.	Date	crude product					pure product				
			Analytical date			Qty.	Yield	Analytical date			Qty.	Yield
			HPLC	LCMS	NMR	g	%	HPLC	LCMS	NMR	g	%
JIBC1111a02-001	JIANL-181-1	11/5/2013								AN130101 3193	1.783	47.2
JIBC1111a02-002	JIANL-184-1	11/11/2013				3.78	100.0					
JIBC1111a02-003	JIANL-189-1	11/21/2013				8.95	100.0					

4.3 Synthesis of BC1111a03

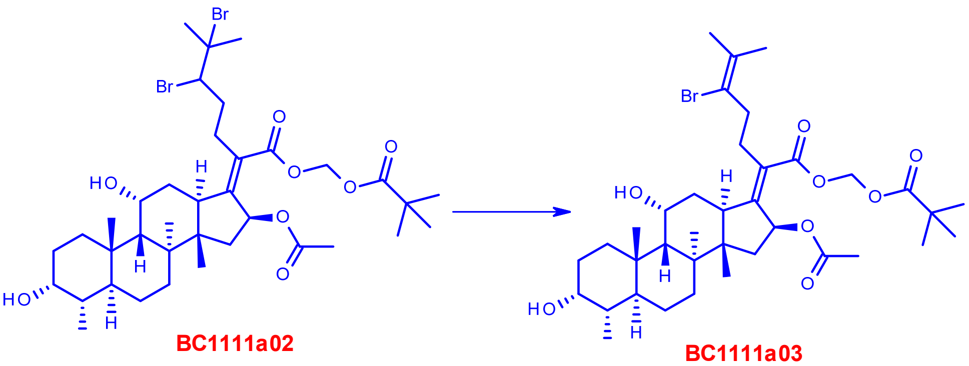

BC1111a02 BC1111a03

Reaction conditions:

Experiment No.	Date	BC1111a02			DBU		CCl_4	Temp.	Time
		Batch No.	g	mmol	g	mmol/eq.	mL	ºC	hr
JIBC1111a03-001	11/5/2013	JIANL-181-1	1.28	1.62	0.49	3.25/2.0	20	77	16
JIBC1111a03-002	11/11/2013	JIANL-184-1	3.78	4.78	1.46	9.56/2.0	50	77	16
JIBC1111a03-003	11/21/2013	JIANL-189-1	8.95	11.3	3.46	22.6/2.0	120	77	16

Results:

Experiment No.	Batch No.	Date	crude product					pure product				
			Analytical date			Qty.	Yield	Analytical date			Qty.	Yield
			HPLC	LCMS	NMR	mg	%	HPLC	LCMS	NMR	g	%
JIBC1111a03-001	JIANL-182-1	11/6/2013					failed					
JIBC1111a03-002	JIANL-185-1	11/12/2013								AN130101 3313	1.29	38.1
JIBC1111a03-003	JIANL-190-1	11/22/2013									3.26	40.6

4.4 Synthesis of BC1111a04

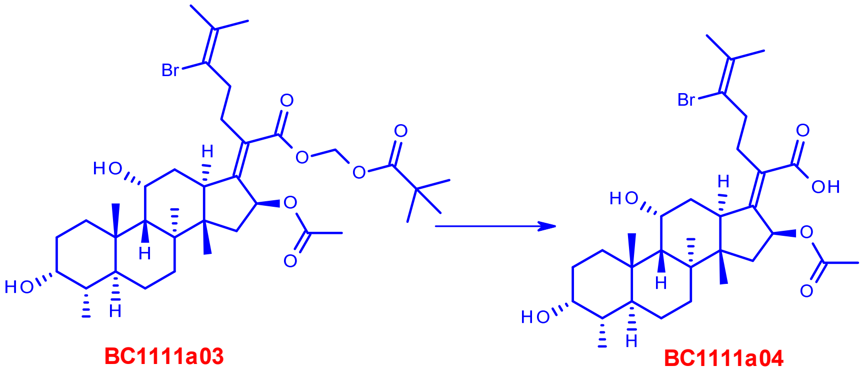

BC1111a03 BC1111a04

Reaction conditions:

Experiment No.	Date	BC1111a03			DBU		MeOH	H_2O	Temp.	Time
		Batch No.	g	mmol	g	mmol/eq.	mL	mL	ºC	hr
JIBC1111a04-001	11/5/2013	JIANL-181-1	0.45	0.63	0.05	0.33/0.53	7.7	4	20	4
JIBC1111a04-002	11/13/2013	JIANL-185-1	1.23	1.74	0.12	0.80/0.46	22.5	12.5	20	4
JIBC1111a04-003	11/21/2013	JIANL-190-1	3.26	4.59	0.32	2.11/0.46	65.0	25.0	20	6

Results:

Experiment No.	Batch No.	Date	crude product					pure product				
			Analytical date			Qty.	Yield	Analytical date			Qty.	Yield
			HPLC	LCMS	NMR	g	%	HPLC	LCMS	NMR	g	%
JIBC1111a04-001	JIANL-183-001	11/7/2013					failed					
JIBC1111a04-002	JIANL-186-001	11/14/2013		AN130339 6701	AN130101 3347	1	68.9		AN130339 7045		0.15	14.5
JIBC1111a04-003	JIANL-191-001	11/25/2013				2				AN130101 3596	0.27	9.9

4.5 Synthesis of BAL0117224 (BC1111)

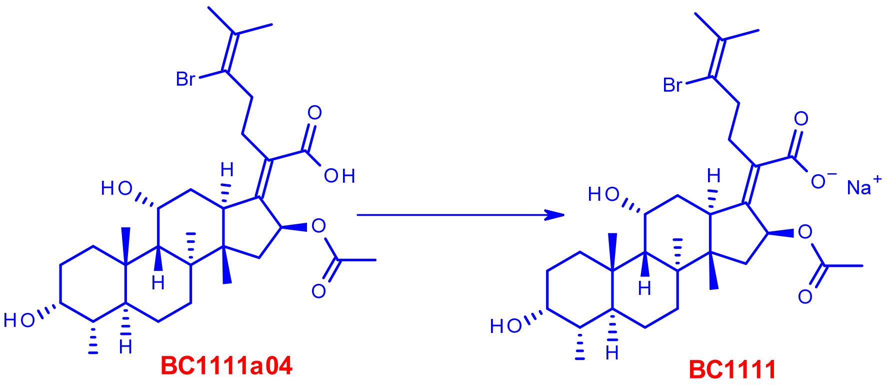

Reaction conditions:

Experiment No.	Date	BC1111a04			NaOH		MeOH	H_2O	Temp.	Time
		Batch No.	mg	mmol	mg	mmol/eq.	mL	mL	ºC	hr
JIBC1111-001	12/4/2013	JIANL-191-1	80	0.13	5	0.13/1.0	1.0	0.134	20	0
JIBC1111-002	12/6/2013	JIANL-191-1	190	0.32	13	0.32/1.0	2.4	0.319	20	0

Results:

Experiment No.	Batch No.	Date	crude product					pure product				
			Analytical date			Qty.	Yield	Analytical date			Qty.	Yield
			HPLC	LCMS	NMR	mg	%	HPLC	LCMS	NMR	mg	%
JIBC1111-001	JIANL-199-001	12/4/2013						AN130221 2940	AN130303 0276	AN130102 5428	67	80.7
JIBC1111-002	JIANL-206-001	12/6/2013						AN130221 3052	AN130303 0304	AN130102 5575	152	77.2
